# CGeNArate: a sequence-dependent coarse-grained model of DNA for accurate atomistic MD simulations of kb-long duplexes

**DOI:** 10.1093/nar/gkae444

**Published:** 2024-05-30

**Authors:** David Farré-Gil, Juan Pablo Arcon, Charles A Laughton, Modesto Orozco

**Affiliations:** Institute for Research in Biomedicine (IRB Barcelona), The Barcelona Institute of Science and Technology, Baldiri Reixac 10-12, E-08028 Barcelona, Spain; Institute for Research in Biomedicine (IRB Barcelona), The Barcelona Institute of Science and Technology, Baldiri Reixac 10-12, E-08028 Barcelona, Spain; School of Pharmacy and Biodiscovery Institute, University of Nottingham, University Park, Nottingham NG7 2RD, UK; Institute for Research in Biomedicine (IRB Barcelona), The Barcelona Institute of Science and Technology, Baldiri Reixac 10-12, E-08028 Barcelona, Spain; Department of Biochemistry and Biomedicine, University of Barcelona, E-08028 Barcelona, Spain

## Abstract

We present CGeNArate, a new model for molecular dynamics simulations of very long segments of B-DNA in the context of biotechnological or chromatin studies. The developed method uses a coarse-grained Hamiltonian with trajectories that are back-mapped to the atomistic resolution level with extreme accuracy by means of Machine Learning Approaches. The method is sequence-dependent and reproduces very well not only local, but also global physical properties of DNA. The efficiency of the method allows us to recover with a reduced computational effort high-quality atomic-resolution ensembles of segments containing many kilobases of DNA, entering into the gene range or even the entire DNA of certain cellular organelles.

## Graphical Abstract

**Graphical Abstract:**
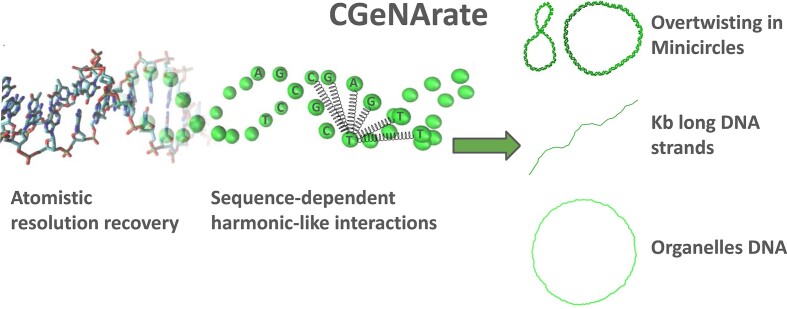

## Introduction

DNA has been both a topic of interest and a challenge for theoreticians who faced the formidable problem of simulating a multiscale system ranging from the base-pair (bp; Å-scale) to the meter-long chromatin fiber of developed organisms (1). At the highest level of resolution, quantum mechanics (QM) theory provides electronic details of small DNA segments (2,3), but most atomistic information of DNA is obtained from the use of molecular dynamics (MD) coupled to classical force-fields (FFs). Last generation DNA FFs (4–6) have achieved a level of accuracy comparable with that of experiments (7), and more impressively, have shown predictive power in a variety of systems, even far from biological conditions (8–12). However, and despite their success, atomistic simulations are limited in the size of the systems to be studied, as the total number of particles to be simulated scales roughly with the third power of the length of the duplex, making in practice impossible to simulate duplexes longer than c.a. 70–100 bp.

Alternatives to atomistic methods aim to reduce the cost of the calculation by using simplified solvent models, merging groups of atoms into beads and representing a Hamiltonian by very simple terms. Two families of approaches have emerged from these ideas: (i) mesoscopic models and (ii) Cartesian coarse-grained approaches. The mesoscopic models take advantage of a helical coordinate system that is the natural one to describe a DNA duplex. In the simplest version, bp step (bps) movements are described as 3 translational (rise, slide, shift) and three rotational (twist, roll, tilt) degrees of freedom, and the energy is computed by using (bps) local harmonic Hamiltonians (13,14), which were fitted to a diverse set of experimentally-determined bps geometries. Second-generation models follow the same physical approaches, but were fitted to atomistic MD simulations (15) which allowed them to be parametrized for all the unique tetramers (i.e. three consecutive bps (16–18)). The latest versions have been extended to capture non-local effects (19–22), non-harmonic deviations (17), and even base pair distortions (20,21,23). All these mesoscopic methods are accurate and computationally efficient, which allow the simulation of medium sized chromatin fibers (24–26). However, they present some intrinsic caveats: (i) no backbone information is directly available from the ensembles; (ii) the mesoscopic methods do not couple well with MD algorithms; finally, (iii) non-bonded terms required to simulate long DNA duplexes are difficult to introduce. Recent approaches based on learning the connection between helical coordinates and backbone geometry in atomistic MD simulations (17) have partially solved the first problem (27), but facing the other two would require a very important development effort, with little guarantee that the resulting method will be still computationally efficient.

Cartesian Coarse-Grained (cCG) models simplify the DNA representation by grouping atoms into beads, whose interactions are treated by simple equations adapted to trace the most usual deformations of DNA. The solvent environment is largely simplified, and sampling is obtained through MD simulations. Broadly speaking, the myriad of cCG models available (reviewed in (1)) can be classified based on: (i) the energy functional, (ii) the number of beads per nucleotide, (iii) the way in which they account for solvent and (iv) the type of strategy used to refine the method. The energy functional can be very different considering the number of beads, the solvent model, and whether cCG is designed to capture near-equilibrium or large denaturing transitions. The number of beads per nucleotide is also very variable: for example, from just 1 bead in Vercauteren's model (28) or MRG-CG (29), 2 in OxDNA (30,31) or Aksimentiev's models (32), 3 of de 3SPN (33), MAD_na_ (34) or BioModi (35) and up to 6–8 beads of high resolution models such as SiRAH (36,37), MARTINI (38), UNRES (39) or HiRe-DNA (40). Despite the reduction in resolution and the large size of the beads, most DNA cCG studies tackle only medium sized (<10^2^ bp) duplexes (1). The treatment of water and ions can be done explicitly (like in MARTINI or SiRAH models) or by means of a continuum model (like in HiRe-DNA or 3SPN, and some versions of SiRAH). Finally, the fitting of functional can follow two main paradigms (which can be combined (1)): (i) the top-down, exemplified by OxDNA or SiRAH, where parameters are refined to reproduce some macroscopic experimental observable, (ii) bottom-up, followed among others by Vercauteren's group or MAD_na_ developers who used atomistic MD simulations as reference. The top-down refinement guarantees accurate average polymer properties, but the lack of enough experimental reference data precludes careful consideration of sequence effects, and no guarantee exists on the accuracy of short-scale details. On the contrary, the bottom-up approach leads to energy functionals that can capture well short-scale details and sequence-dependent effects, but they rely on force-fields whose ability to reproduce polymer properties is not always granted. In summary, there is a plethora of methods available, and the end user should make a careful selection based, mainly, on the nature of the problem.

For biological applications the main challenge of these methods is to reproduce very long segments of DNAs (above kbase), with accurate sequence specificity. Sampling must be fast, but if required, the full atomistic description should be recoverable, allowing detailed representation of DNA interactions. Here we present CGeNArate, a new cCG method created to explore the dynamics of long segments of DNA, approaching those of interest for the representation of chromatin. The method uses implicit solvent, only 1 bead per nucleotide, a simple energy functional including up to 4th order bonded terms, coupled with simple long-range electrostatic and steric functionals. The method, implemented in a ‘de novo’ MD code, can easily manage very long oligomers (above kb scale) and has been parametrized from all-atom MD simulations following a bottom-up approach with a tetramer-level sequence specificity, but taking also into consideration global properties obtained from simulations of long oligomers. It shows an unexpected ability to reproduce mechanical and dynamical properties of a variety of oligomers which were not considered during the parametrization, including circular DNAs, kb long duplexes and even entire mitochondrial DNA. Additionally, the use of a novel machine learning (ML) approach trained with a large dataset of atomistic MD simulations, allows us to map with astonishing accuracy the cCG trajectories into atomistic ensembles for part or the entire duplex. We expect CGeNArate will become a valuable tool to describe segments of the chromatin fiber, even substituting state-of-the-art mesoscopic models(17,19,20,23).

## Materials and methods

CGeNArate is intended to simulate duplex DNA, not extremely far from the equilibrium geometry (as it happens in chromatin). The method uses 1 bead per nucleotide located at the C1’ atom position of the sugar, which facilitates the Machine-Learning back-mapping to the atomistic level and allows a reasonable description of DNA shape.

### Hamiltonian definition

The energy functional is defined as the addition of sequential-dependent (bonded) and remote (non-bonded) terms as described in Eq. ((1)):


(1) \begin{equation*}E = {E}_{seq} + {E}_{remote}\end{equation*}

**Figure 1.**
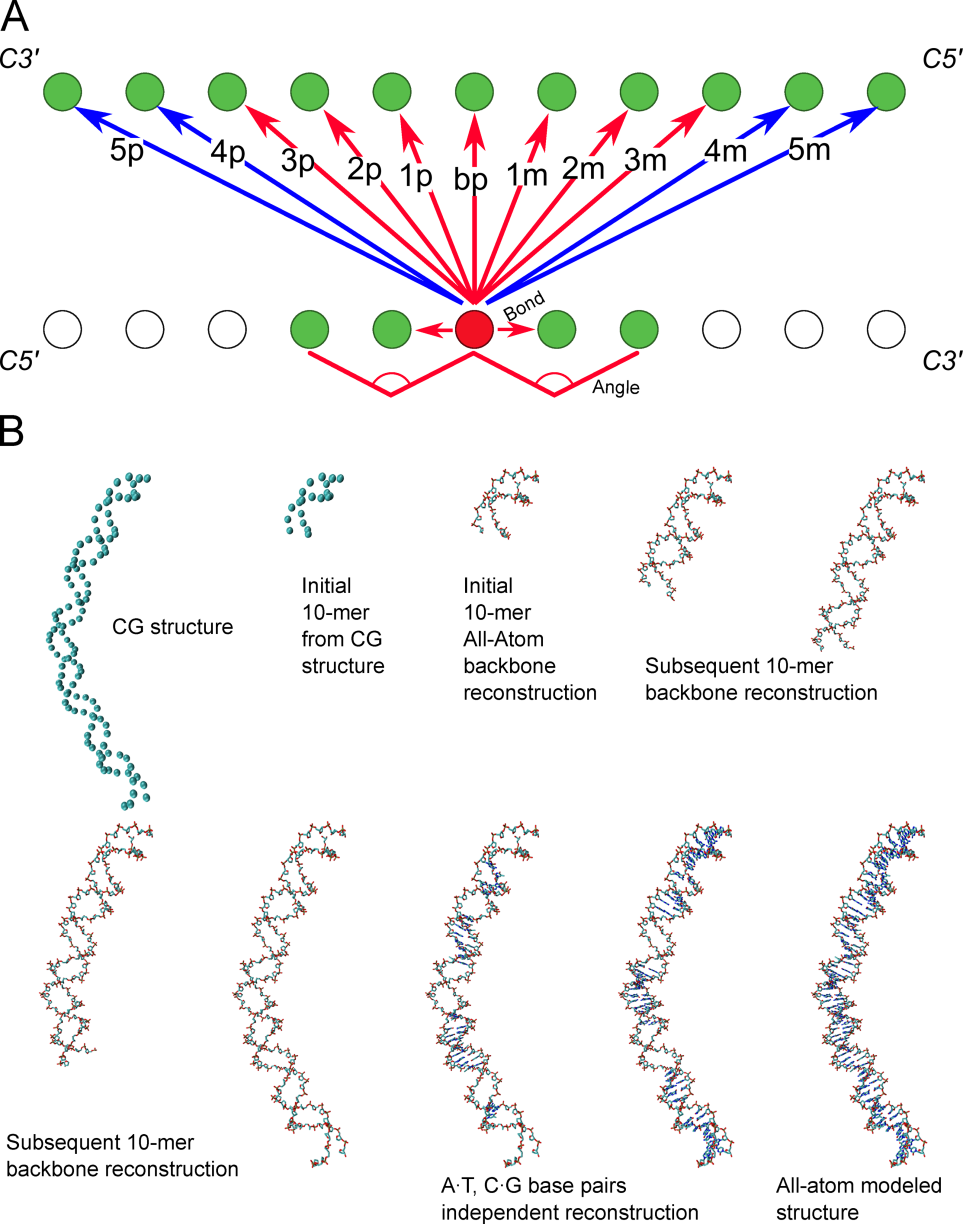
Methods (**A**) outline of the seq terms used in the model. Blue arrows correspond to distant terms, while red arrows represent 4mer terms. (**B**) Step by step evolution of the All-Atom reconstruction process from the CG duplex.

Following Savelyev and Papoian (41), the sequential contribution (${E}_{seq})$ is computed considering 11-bead windows (Figure 1). This means that each bead *i* in the Watson strand (Figure 1) interacts with its neighboring beads (*i* + 1 and *i* −1), with its paired bead (*j*) in the Crick strand, as well as with 5 beads upstream and 5 beads downstream the Crick's paired bead (i.e. *j* + 1 to *j* + 5 in one direction and *j* − 1 to *j* − 5 in the other), and the *i* + 2*i* − 2 bead through angle-dependent interactions (see below). The sequential term is divided into two contributions: one is tetramer dependent $({E}_{seq - 4mer})$ and is calibrated from atomistic MD simulations considering sequence-dependent properties of DNA (see ‘Fitting the Hamiltonian’ section), and the other, which accounts for distant interactions in the 11-bead window$\ ({E}_{seq - distant})$, is calibrated with sequence-averaged dynamic information of DNA (see Figure 1); Eq. ((2)):


(2) \begin{equation*}{E}_{seq} = {E}_{seq - 4mer} + {E}_{seq - distant}\end{equation*}

Note that interactions that are present in two tetramers are obtained by averaging between them (e.g. the angle interaction between beads *i*− 1, *i*, *i* + 1 is shared by the tetramers *i* − 2, *i* − 1, *i*, *i* + 1 and *i* − 1, *i*, *i* + 1, *i* + 2, see below).

Following again Savelyev and Papoian (41), we consider (Figure 1) *stacking* interactions (i.e. *i* : *i* + 1 and *j* : *j* − 1) and *angle* interactions (*i* : *i* + 1 : *i* + 2 and *j* : *j* − 1 : *j* − 2), affecting both Watson and Crick strands and the cross-interactions: *pairing* (*i* : *j*) and *fan* (*i* : *j*− 1, …, *i*: *j* − 5 and *i* : *j* + 1,…, *i* : j + 5). Each interaction is represented by a truncated polynomial expansion. The 2nd order term introduces a basal harmonicity which is modulated by the 3rd order term, while the 4th order term avoids large unrealistic distortions that might happen under stress conditions; see Eq. ((3),(4)).


(3) \begin{equation*}{E}_{stacking,pairing,fan} = \mathop \sum \limits_{a = 2}^4 {K}_a{\left( {l - {l}_0} \right)}^a\end{equation*}

(4) \begin{equation*}{\mathrm{\ }}{E}_{angle} = \mathop \sum \limits_{a = 2}^4 {K}_a{\left( {\alpha - {\alpha }_0} \right)}^a\end{equation*}

where *K_α_* are the force constants of the interaction, *l*_0_ is the equilibrium distance between beads and *α*_0_ is the equilibrium angle (see ‘Fitting the Hamiltonian’ section for details on how they are derived). Note that, by construction, large deformations leading to base opening or kinks in the fiber are not allowed, but further versions of the method, where harmonic terms would be substituted by Morse-like potentials could account for this type of extreme deformation.

Thus, ${E}_{seq - 4mer}$ is determined as shown in Figure 1 and Eq. ((5)):


(5) \begin{eqnarray*}{E}_{seq - 4mer} = \mathop \sum \limits_{Tetramer} \left( {\mathop \sum \limits^2 {E}_{stacking} + \mathop \sum \limits^2 {E}_{pairing} + \mathop \sum \limits^4 {E}_{angle} + \mathop \sum \limits^8 {E}_{fan}} \right)\nonumber\\ \end{eqnarray*}

with the sequential long-term interaction defined as the remaining sequential interactions (see Figure 1), as described in a compacted form in (6)):


(6) \begin{equation*}{E}_{seq - distant} = \mathop \sum \limits_{\begin{array}{@{}*{1}{c}@{}} {index = }\\ { - 5, - 4,4,5} \end{array}} \left( {\mathop \sum \limits_i {E}_{fan}} \right)\end{equation*}

The remote term is divided as follows:


(7) \begin{equation*}{E}_{remote} = {E}_{LJ} + {E}_{ele}\end{equation*}

For further details on the remote terms, see Supplementary Methods (Remote term specifications). Note that to avoid double counting interactions, the ‘remote contribution’ is switched off for interactions between the neighboring beads within 5 bp in both strands (see Figure 1). Note also that alternative formalisms can be implemented to account for intermolecular interactions involving charged polyelectrolytes (42).

### Fitting the Hamiltonian

The sequential tetramer (seq-4mer) parameters were refined in an iterative manner, taking equilibrium distances and angles from atomistic MD simulations. A first set of sequence-independent parameters was obtained by fitting force-constants in Eq. (4) to reproduce as close as possible variances and covariance of the different distances and angles included in the Hamiltonian definition. We perform then a 1st tetramer-based parametrization, where the *seq-4mer* terms are refined sequentially for each tetramer in the context of initial guesses for the parameters of the remaining tetramers (average parameter of all tetramers). Once 2nd iteration parameters are obtained for all the tetramers in the sequence, the process is repeated until convergence is achieved (typically, 3–4 iterations are required for convergence). Data used for fitting was obtained from the thirteen 18-mer duplexes of the miniABC dataset (16) stored in the BigNAsim database (mmb.irbbarcelona.org/BIGNASim (43)). Parameters were refined using the constrained optimization by linear approximation (COBYLA) method (44), which allowed us to avoid overtraining artifacts that would lead to physically unrealistic parameters. (see Supplementary Methods Parameter fitting at tetranucleotide level). The overall optimization process leads to a significant improvement in the overlap between the distribution of CG and AA observables (see Supplementary Figure S1)

The *seq-distant* terms (*i*→*j* ± 4 and *i*→*j* ± 5) were obtained initially from equilibrium values of atomistic MD simulations, and then re-adjusted by fitting a 40-mer duplex. Standard annealing procedures increasing and decreasing the ‘effective temperature’ was used to refine the associated parameters, accordingly Metropolis-Hasting simulations with different seeds were computed and the best of the sampled sets were refined by conjugate gradient minimizations. The end-to-end distance and the associated variance were used as merit functions in the fitting. The remote non-bonded electrostatic term (Eqs. (8) and (9)) was determined considering *q* = −1 in each bead, and dielectric and inverse distance parameters corresponding to 100 mM NaCl aqueous solution. Following previous works (17,45) Lenard Jones parameters $\sigma$ and ${\epsilon }_{LJ}$ were set to 10 Å and 0.59 kcal/mol.

### Integration of the equations of motion

The Hamiltonian above has been implemented in a *de novo* Langevin Dynamics code. Integration of the equations of motions was performed using velocity Verlet with an integration step of 0.1 ps, which guarantees stability in the trajectory for temperatures up to 500 K (see Supplementary Figure S2). The masses of the beads correspond to those of the nucleotides. Temperature was maintained constant using Langevin bath with standard coupling parameters (46). Friction terms corresponding to those of Brownian stochastic forces were generated following a Box–Muller transformation (47). As noted in Supplementary Figure S2, the method is quite robust to small perturbation of these parameters.

### All atom rebuilding

The 1-bead per nucleotide trajectories were back-projected to all-atom resolution by using a machine learning (ML) approach that takes the CG coordinates through time and the sequence of the duplex as descriptors. The method is developed from the GLIMPS (27) approach, which was originally created to rebuild atomistic structures from mesoscopic descriptors. As shown in Figure 1, atomic resolution back-projection was done in two steps: (i) backbone reconstitution and (ii) generation of the A·T/T·A and G·C/C·G geometries. In all cases, training was done for all the 10-mer contained in 40-mer trajectories deposited in our BigNAsim database (43). The all (heavy) atom Cartesian coordinates were the objective values, while the C1’ CG coordinates (from the same atomistic simulations) and the duplex sequence were the descriptors.

Once training is done, all atoms reconstitution is performed using in general 10-mer blocks. As described above, we first use one GLIMPS model to rebuild the sequence-neutral backbone atom positions for the decamer segment from the C1’-atom positions, then use a second, base-pair dependent, GLIMPS model at each of the 10 base pair steps to rebuild the base atom positions from the now-established sugar and phosphate positions. Finally, as some local distortions appear, a short steepest descent minimization is performed using the ParmBSC1 force-field. Structural changes introduced by these geometry optimization steps are small (typically tenths of Å), but steric clashes that might appear during the coarse-grained to all-atom decoding are removed (see Figure S3).

### Validation analysis

All validation analysis was performed using different sequences to those used for the training of the method and using when possible both MD-derived and experimental values. Details of the different sequences and metrics used for validating the ensembles are detailed in Supplementary Material.

### Computational details

All-atom simulations were performed using PARMBSC1 force-field (4), explicit solvent (SPC/E (48) or TIP3P (49) water models) and 100–200 mM salt using state of the art simulation conditions (16,50) at room temperature and pressure (see Supplementary Information for additional details). Trajectories are stored in our BigNAsim database (43). For comparison purposes, additional simulations were done using implicit solvent MD, using standard AMBER GB/SA implementation ((51); additional details in Supplementary Information).

## Results and discussion

### The coarse-grained trajectories

**Figure 2.**
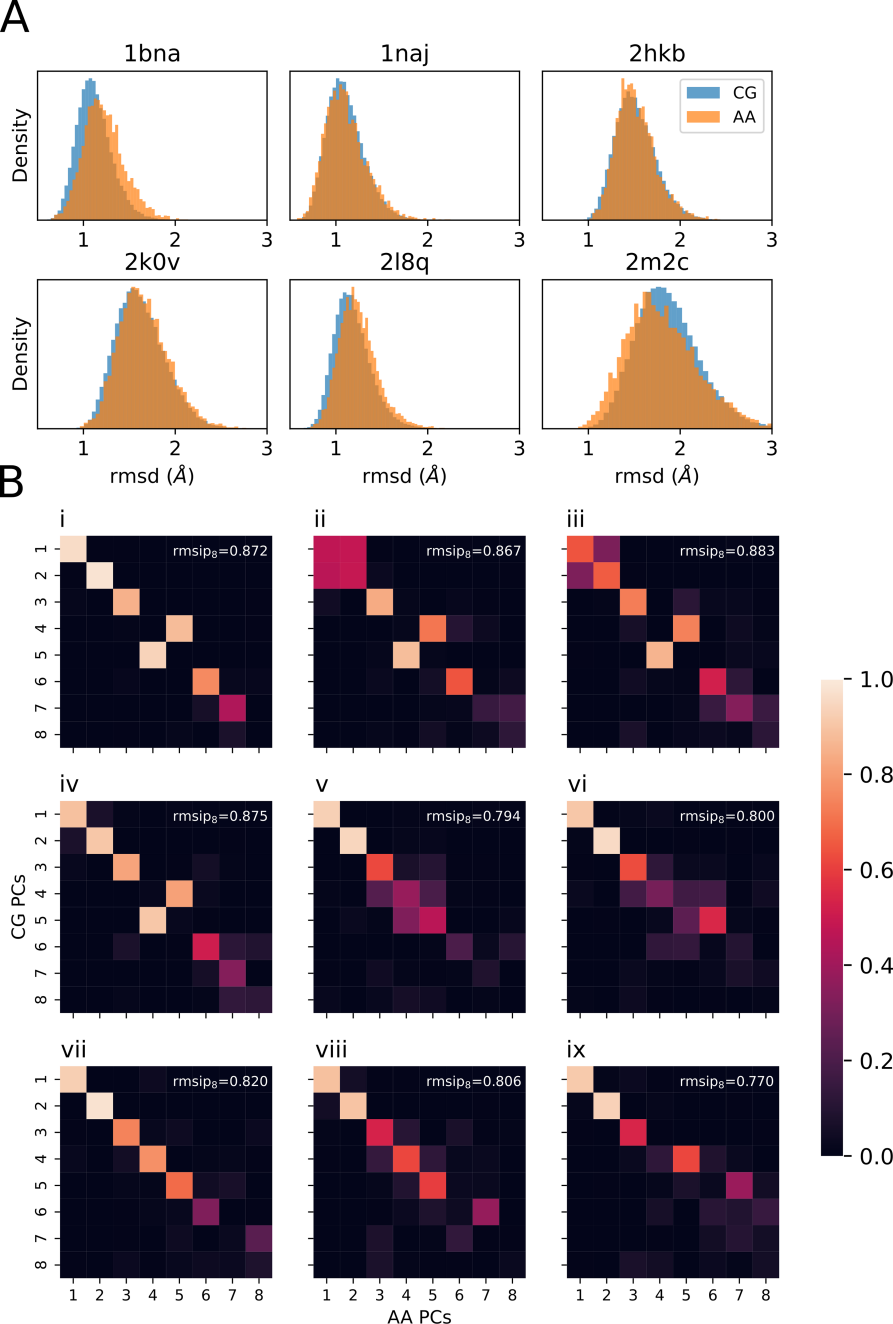
Evaluation metrics of the Coarse-Grained trajectories: (**A**) rmsd distribution between C1’ atoms of simulated Coarse-Grained (CG, blue) and all-atom (AA, orange) trajectories against the pdb experimental reference structure indicated in each panel. (**B**) Root mean square inner product (rmsip) between CG and AA principal components of trajectories from the test set, ordered by decreasing variance. Each panel corresponds to simulations of the following structures (see Materials and methods). A: BigNAsim Code CGTG, B: BigNAsim Code AGCT, C: 1zgw, D: BigNAsim Code AGCG, E: BigNAsim Code CTAG_flex, F: 2lef, G: 1j5n, H: 2m2c, I: 1naj.

The CG method and its associated Hamiltonian presented here can provide ensembles which resemble very closely at the C1’ level those derived from atomistic MD simulations obtained with *state-of-the-art* force-fields and explicit solvent representation (AA-trajectory). For the 18-mer duplexes included in the miniABC database(16) the RMSd (C1’) between all-atom and CG MD averaged structures is 0.79 Å (i.e. 0.04 Å × bp), a very small value, within the standard deviation implicit to the average (around 1.7 Å). Note that this good fitting is remarkable considering that the training was done at the 4-mer level, not for the entire duplex, whose global structure was not considered at any point of the calibration. Even more impressively: the results obtained for duplexes out of the training set are also very accurate, not only in terms of the reference AA MD trajectory, but also of experimental structures (see Figure 2). Interestingly, the histograms of RMSd are quite similar in CG and AA simulations, suggesting that the model captures flexibility well (Figure 2). This is confirmed by the inspection of essential deformation modes, which are almost identical in CG and AA simulations as noted in the global overlap between the first ten modes around 0.9 for the miniABC dataset (see Table 1 and selected examples of eigenvectors overlap in Supplementary Figure S4). The performance is maintained for duplexes not considered in the training process (Figure 2), which demonstrates that the essential deformation movement of DNA is very well recaptured by our method (see Supplementary Figure S5). Global flexibility descriptors such as the persistence length, or end-to-end distance (see Supplementary Methods) are also correct (see Table 1), fitting in fact better experimental values than the reference AA values, something that was also found in mesoscopic models of DNA (42). This probably occurs because of some fortuitous error cancellation related to the neglect of long-range anti-correlation effects that corrects a tendency of PARMBSC1 to overestimate DNA stiffness. Finally, and very encouragingly, the model can accurately capture sequence variability. This is shown in Figure S6, where the cross RMSds between 500 structures (CG or AA) of the thirteen 18-mer duplexes (i.e. 13 × 500 total structures) are reported. Not only are the lowest RMSd obtained along the diagonal, reproducing the AA simulations, but even the out of the diagonal similarities detected in AA-simulations are well reproduced in our CG simulations. This indicates that the model is reproducing sequence-dependent structural details with a quality similar to AA simulations. Note again that no training was done using global structural parameters of the duplex.

**Table 1.** Global deformation properties of the 13 sequences of the miniABC dataset (16)

	1	2	3	4	5	6	7	8	9	10	11	12	13
RMSIP AA to CG	0.87	0.87	0.86	0.89	0.86	0.87	0.88	0.86	0.88	0.88	0.88	0.87	0.87
PL AA	58	58	62	67	61	72	66	64	68	56	57	60	60
PL CG	50	50	59	52	55	64	67	56	62	52	49	54	54
EtE AA	56	56	57	56	57	57	57	56	57	57	57	57	56
Std	1.6	1.7	1.6	1.6	1.6	1.6	1.5	1.6	1.5	1.6	1.8	2.0	1.6
EtE CG	58	57	59	58	59	58	59	58	58	58	58	59	58
std	2.0	2.0	1.8	1.9	1.9	1.8	1.8	2.0	1.8	2.0	1.9	1.9	2.0

Global deformation properties of CG simulations.

RMSIP is the inner product between the all-atom (AA) and the coarse-grained (CG) trajectories. PL stands for the persistence length (in nm), EtE is the end-to-end distance (in Å) in AA or CG simulations with std being the associated standard deviation (another proxy of flexibility).

### The AA reconstituted trajectories

**Figure 3.**
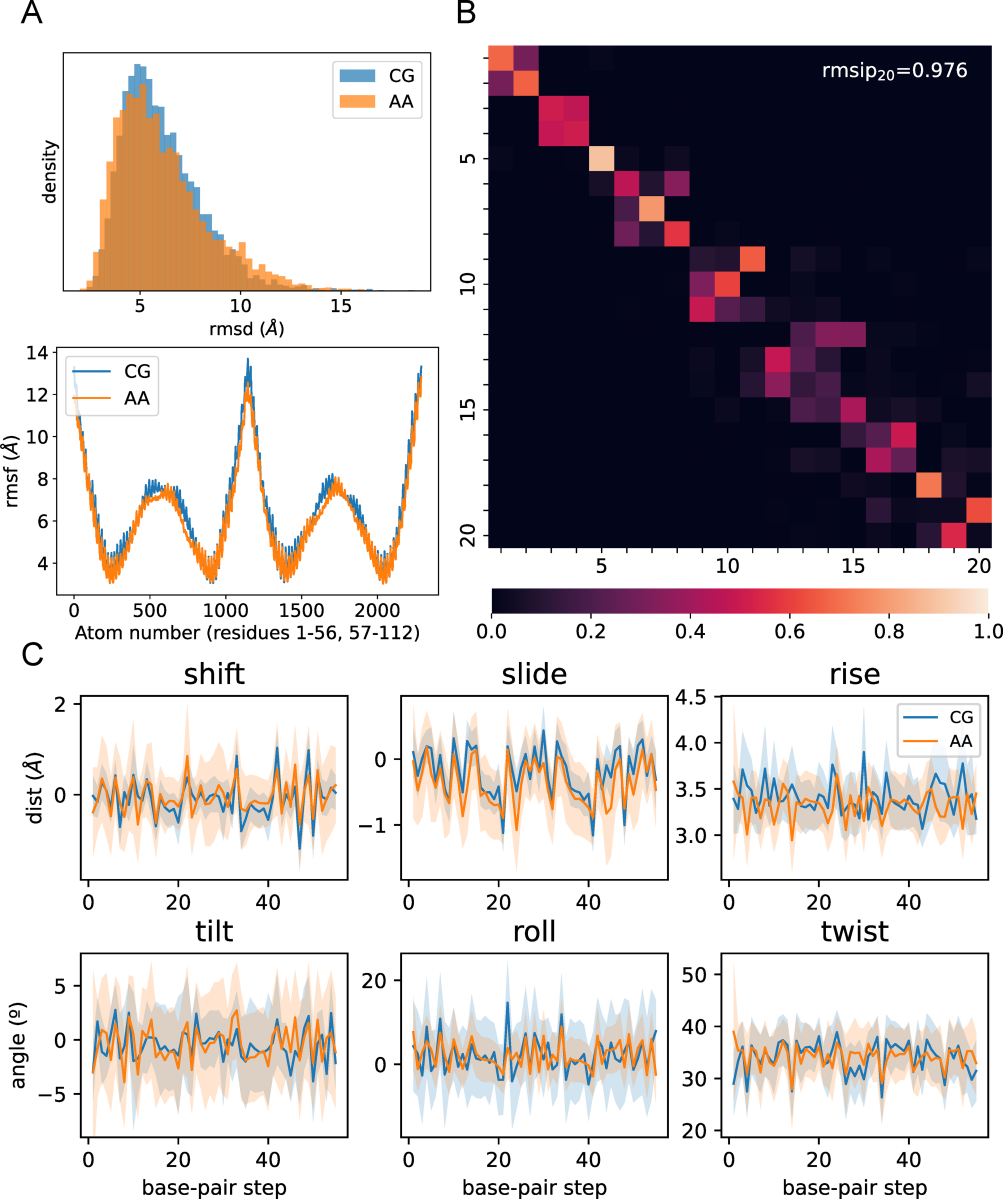
Results from the 56-mer simulations. (**A**) (Top) All-Atom RMSD distribution against AA average from the reconstructed CG structures (blue), and from the AA simulation (orange). (Below) Mean fluctuation per atom of reconstructed CG simulation (blue), and the AA simulation (orange). The atom number ranges through one strand in 5′-to-3′ direction, then the other strand in the same direction. (**B**) Root mean square inner product matrix between CG and AA principal components extracted from the respective trajectories. (**C**) Helical parameters across each base-pair step for both CG and AA trajectories. Lines represent mean values, and shadows represent 1 standard deviation.

When the decoding process is performed, the all-atoms reconstituted trajectories are surprisingly close to the original all-atom (AA) trajectories (all heavy atoms RMSd) around 0.102 Å per bp in the mini-ABC database and around 0.095 Å per bp for the different duplexes considered for validation set. The analysis of a 56-mer duplex available in the BigNAsim database (diverse in composition and 3 times larger than the mini-ABC duplexes) demonstrate that the reconstituted trajectory (decoding the CG ensemble) and the AA trajectory (not considered at any point during the calibration of the model) are hard to distinguish (Figure 3, Supplementary Figure S7), not only in terms of general structural descriptors, but also of atomic fluctuations, essential deformation modes, and sequence-dependent helical parameters. A more in-depth exploration of Atomistic details, helical and backbone structures is provided in SI (Supplementary Figures S8 and S9), as well as further testing of helical parameters on a range of sequences (Supplementary Figures S10 and S11). Results suggest a quality beyond the expectations of a C1’-only model, which indicate that some fine details such as backbone geometries are somehow (at least partially) captured in the C1’ geometries in a way that ML approaches can capture them. The most sizeable differences between AA and CG distribution is for the standard deviations of some helical parameters (see Figure 3), indicating the intrinsic shortcomings of the C1’ representation. Correction of these deviations would require the addition of extra degrees of freedom.

### Circular DNAs

**Figure 4.**
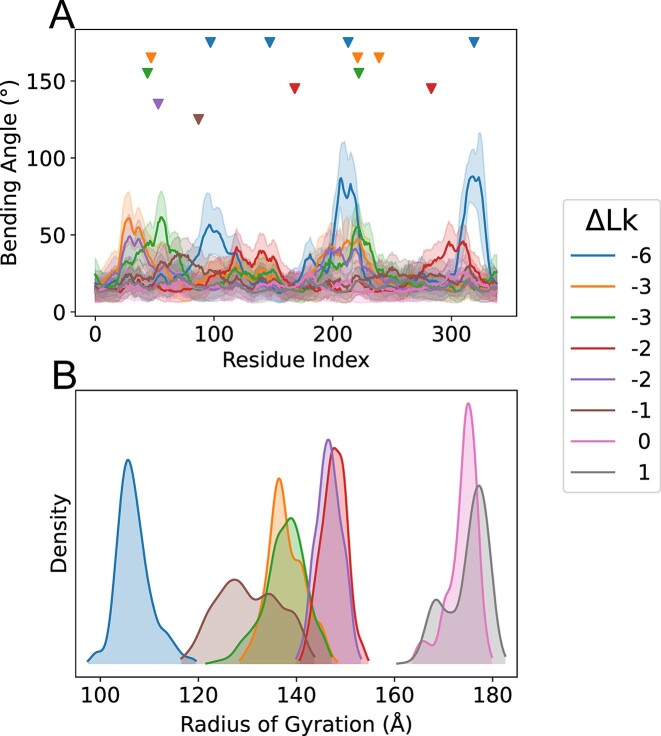
Results from simulations of Circular DNA. (**A**) Bending Angle per base pair from CG simulations, colored by Linking Number difference (ΔLk). Lines represent mean values, and shadows represent 1 standard deviation. Triangles at the top represent kinks/defects in the Atomistic simulations, with the same color coding. (**B**) Radius of Gyration distribution from the CG Simulation, colored by linking number difference.

In order to test the limits of our model we explored 339 bp circular DNA, which has been already studied by AA MD simulation, and for which electron microscopy images are available(52). Starting structures (see Supplementary Methods for generation scripts) from relaxed DNA quickly converged into the predicted supercoiling, leading to stable trajectories sampling a wide range of conformational space. By construction, the method is not able to capture kinks, but those regions where kinks appear in atomistic MD simulations (52) (for a given supercoiling) are those where higher bending angles are obtained (Figure 4), showing the ability of the model to identify softer and more flexible regions of the minicircle. The Global descriptors captured from CG simulations show diversity in structure, related to those collected from electron microcopy measures (see Figure 4, Supplementary Figure S12 and reference (52)) and a wide sampling of conformational shapes are obtained as visible in the oscillation of the radii of gyration along the trajectory (see Supplementary Figure S12). Finally, and quite surprisingly, the ML method, which was trained with linear DNA, maintains a good ability to back-map minicircle trajectories to AA resolution (see Supplementary Figure S12). In summary, our CG model and associated ML-reconstitution algorithm are not optimal to explore extremely stressed DNAs like those in minicircles, where the elastic regime might not be valid, but they can be very useful to perform massive screenings to obtain reasonable atomic resolution ensembles from which AA MD simulations can be performed.

### Very long systems

**Figure 5.**
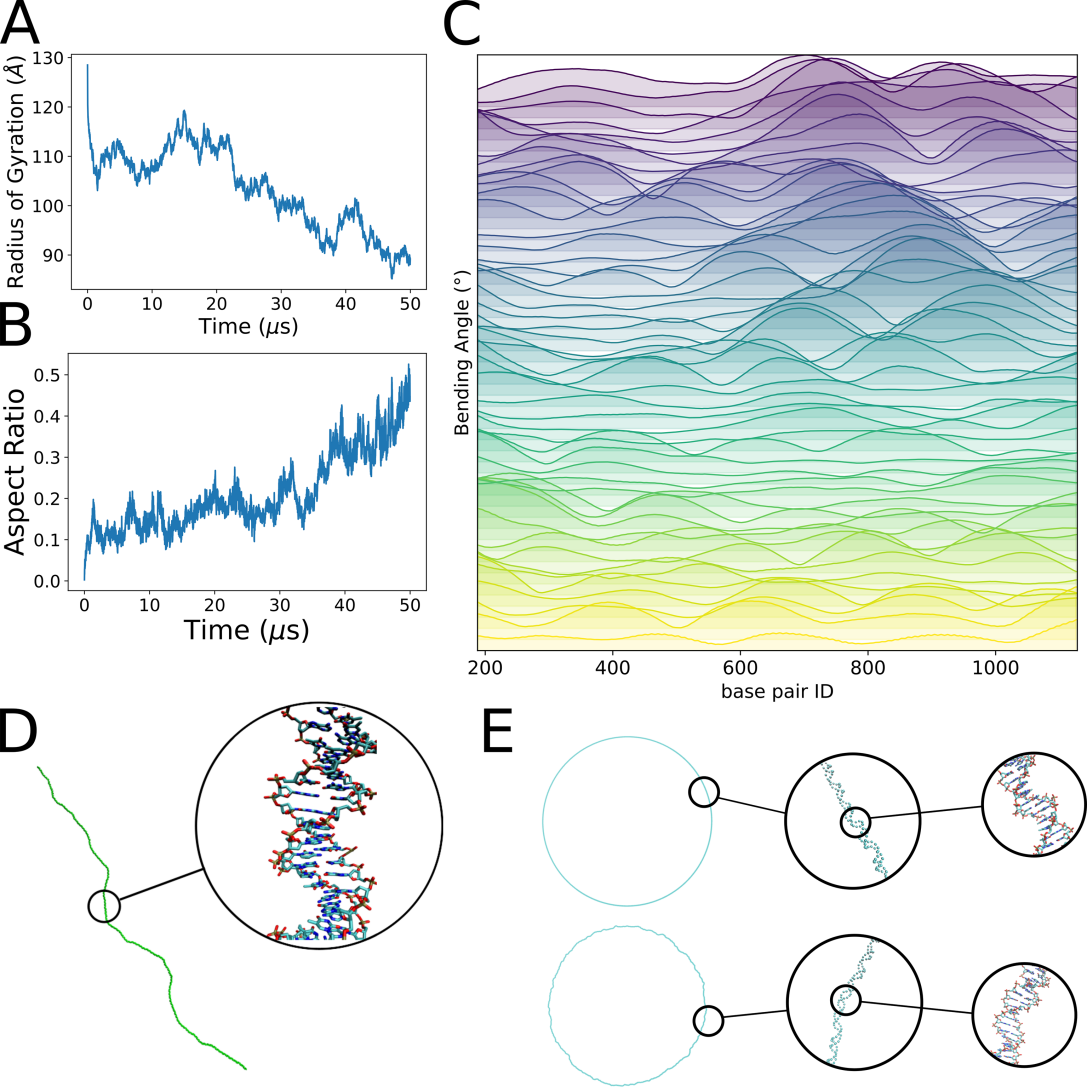
Coarse-grained simulation of yeast gene YCL020W containing 1317 bp, and Mitochondrial DNA. (A, B) Time evolution of the radius of gyration (**A**) and the aspect ratio (**B**) of the structure of the gene. (**C**) Bending angle per base pair of the gene structure, computed with a window of 196 bp, colored by simulation fragment, first plot corresponds to the first microsecond of simulation, second corresponds to the second microsecond and so on. The values correspond to the average over each trajectory block. Plots go from bottom (yellow) to top (purple), as the simulation blocks increase from block 1 to block 50. (**D**) Representative snapshot showing the all-atom reconstruction of the gene structure. (**E**) Human mitochondrial DNA structure at three different scales. Zoom at the top corresponds to a frame near the beginning of the simulation. Zoom at the bottom is after 15 μs of simulation, where local distortions are evident.

The objective of any CG representation of DNA is to expand the size of the systems accessible to simulation. We tested here the performance of the method in two large systems: i) a 1317-mer long duplex (2.6 kbases in terms of mass) (link, Supplementary Movie S1) bearing the YCL020W gene of *Saccharomyces cerevisiae* (the TYA retrotransposon coding for the TY1 virus-like particle) and ii) the human mitochondrial DNA (33 kbases) (link, Supplementary Movie S2). Any of these two systems is very far away from what is accessible to atomistic MD simulations, but can by simulated by our model even when using a single processor desktop computer, providing clues of the DNA flexibility in the polymeric range, which to our knowledge, were never described before. Figure 5 shows details of a 50-microsecond simulation of YCL020W gene. It is clear that starting from an unrealistic straight DNA the trajectory moves to a more compact and curved structure (around 20% decrease in radii of gyration) in the 2–3 microsecond range to oscillate quite periodically along the trajectory. Analysis of the bending angle (196 bp window; see Figure 5) illustrates how the local curvature is propagated along the sequence, suggesting the existence of a perturbation dynamics propagation mechanism (53), which also agrees with the structural diversity found in atomic force microscopy experiments (54). Finally worth to note that trajectory is still not converged, but a parallel version of the code would allow reaching the multi-millisecond time scale, where convergence is expected.

As a final test model, we simulate the entire human mitochondrial DNA (Figure 5) at Lk = 0. This system, a double stranded circular DNA containing more than 16 kb, encodes ribosomal and transfer RNA as well as up to 13 proteins, whose malfunction is linked to different rare diseases. The mitochondrial DNA is found in nucleoids resembling the prokaryotic ones, probably mimicking phase separation in eukaryotic chromatin. Proof of concept simulations under high salt conditions, even with relatively short times of 15 μs, show deviations from perfect circularity that are transferred along the sequence, mimicking linear DNA. Analysis of relative end-to-end distance and bending angle of the longest coding genes shows the existence of a quite variable geometrical landscape (see Supplementary Figure S13). Simulations are too short to guarantee the convergence of the results, but illustrate the power of the method to analyze complex chromatin in detail.

### Reproducing elastic properties of repetitive sequences

**Figure 6.**
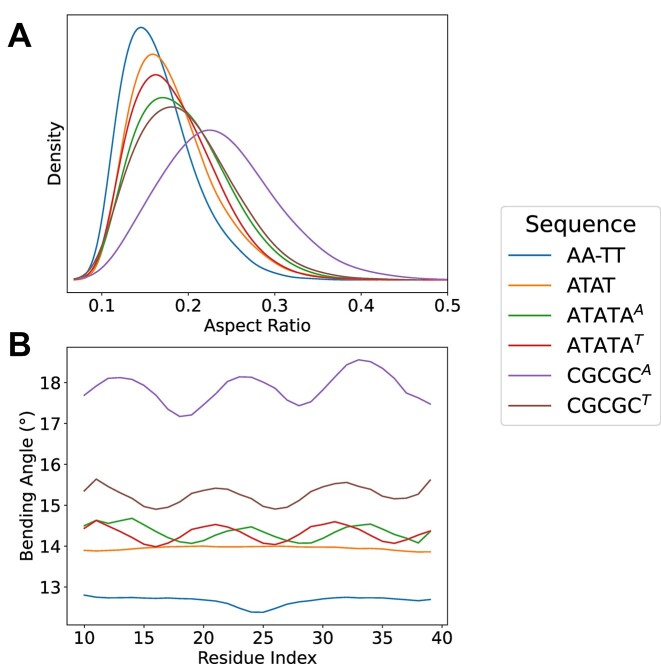
(**A**) Distribution of the aspect ratio and (**B**) mean local bending angle along the sequence of the duplex DNA (see Supplementary Methods for description of the different metrics) for 50-mers with repetitive sequences: AA-TT AAAAAAAAAAAAAAAAAAAAAAAAATTTTTTTTTTTTTTTTTTTTTTTTT. ATAT ATATATATATATATATATATATATATATATATATATATATATATATATAT, TATA^A^**ATATA**AAAAA**ATATA**AAAAA**ATATA**AAAAA**ATATA**AAAAA**ATATA**AAAAA, TATA^T^**ATATA**TTTTT**ATATA**TTTTT**ATATA**TTTTT**ATATA**TTTTT**ATATA**TTTTT, CGCG^A^**CGCGC**AAAAA**CGCGC**AAAAA**CGCGC**AAAAA**CGCGC**AAAAA**CGCGC**AAAAA, CGCGC^T^**CGCGC**TTTTT**CGCGC**TTTTT**CGCGC**TTTTT**CGCGC**TTTTT**CGCGC**TTTTT.

The method was parametrized to reproduce tetramer-based properties of DNA as determined by atomistic MD simulation, but quite encouragingly, it shows a good ability to reproduce the properties of repetitive sequences where sequence-dependent effects are maximized. For example, the AA-TT oligo is expected to be straight and rigid(55) with a discontinuity in the central A-T step, which is exactly what is found in our simulations (Figure 6). Similar behavior is expected for the ATAT oligo, where symmetry hinders any local fluctuation leading to a very rigid and straight segment, as found in our calculations (see Figure 6). Periodic oscillations in bending should be recovered when symmetry is broken and the ATATA segment is placed in phase (every 10 bp) separated by A_5_ or T_5_ linkers, as again it is found in our calculations (Figure 6). Finally, when a more flexible segment such as CGCGC(16,55,56) is placed in phase, separated by A_5_ or T_5_ linkers, large fluctuation in bending can be expected with maxima of bending at the flexible segments, as is in fact shown (see Figure 6). So, despite the simplicity and locality of the model, it is able to reproduce well the expected behavior of repetitive sequences.

### Computational performance

The method is very efficient allowing the simulation of extended DNA duplexes for significant periods of time. Results (see Supplementary Figure S14.A) indicate that in just one CPU hour, 10 ns of CG simulations can be obtained for a 1317-mer duplex (corresponding to one entire Yeast Gene), while we expect simulation times for atomistic MD simulations to be at least 7 orders of magnitude longer. The cost of the decoding process to generate atomistic trajectories is very small, for example, reconstituting 5000 snapshots of the 1317-mer duplex implies 20 min of CPU, keeping the simulation cost nearly unaltered. Note that the method is superior to implicit solvent GBSA, not only in terms of computational efficiency, but also of quality of the ensembles. Clearly atomistic/explicit solvent and CG simulations are indistinguishable, while GB/SA diverges, leading to very close CG and AA structures, while showing a very deformed GB structure (see Supplementary Figure S14B, C).

## Conclusion

We present here a new sequence-depended coarse-grained method which, thanks to an associated ML algorithm, provides atomistic resolution trajectories difficult to distinguish from ‘state of the art’ all-atom simulations, but with a significant reduction of computational effort. The method (publicly available at gitlab) enables the dynamic study of large segments of chromatin, covering entire genes and providing dynamic information with an unprecedented level of quality and resolution. Our results pave the path to a new generation of high-quality modeling tools exploring the dynamics of large chromatin segments coupled to change in the environment, presence of ligands, protein effectors, or even condensation phenomena.

## Supplementary Material

gkae444_Supplemental_Files

## Data Availability

The data that supports the findings of this study is openly available in the public repository https://mmb.irbbarcelona.org/gitlab/dfarre/cgenarate-materials. The executable for CGeNArate is also available in the public repository https://mmb.irbbarcelona.org/gitlab/dfarre/cgenarate-materials. Simulation files will be deposited in BigNAsim (https://mmb.irbbarcelona.org/BIGNASim/). The list of accession numbers is provided in the Supplementary Data.
