# Supplementary material for: CGeNArate: a sequence-dependent coarse-grained model of DNA for accurate atomistic MD simulations of kb-long duplexes
Source: Nucleic Acids Res. 2024 May 30;52(12):6791–801. doi: 10.1093/nar/gkae444 (PMC11229373; doi:10.1093/nar/gkae444)
Supplement: gkae444_Supplemental_Files [file gkae444_supplemental_files.zip › AccessionNumbers.docx]

**miniABC sequences**

- **1µs (frames every 20ps) for each of the 13 miniABC sequences (2)**

NAFlex_miniABC_1_CG, NAFlex_miniABC_2_CG, NAFlex_miniABC_3_CG, NAFlex_miniABC_4_CG, NAFlex_miniABC_5_CG, NAFlex_miniABC_6_CG, NAFlex_miniABC_7_CG, NAFlex_miniABC_8_CG, NAFlex_miniABC_9_CG, NAFlex_miniABC_10_CG, NAFlex_miniABC_11_CG, NAFlex_miniABC_12_CG, NAFlex_miniABC_13_CG

**Sequences used for validation**

- **1µs (frames every 20ps) for each of the 14 sequences used for validation (as described in Supplementary methods)**

NAFlex_56merL_CG, NAFlex_CGTG_CG, NAFlex_AGCT_CG, NAFlex_AGCG _CG, NAFlex_CTAG_CG, NAFlex_2lef_CG, NAFlex_1j5n_CG, NAFlex_1zgw_CG, NAFlex_DtipDang015M_CG, NAFlex_DDD_II_1_CG, NAFlex_2hkb_CG, NAFlex_2k0v_CG, NAFlex_2l8q_CG, NAFlex_2m2c_CG

**Circular**

- **1µs (frames every 20ps) for Circular DNA with different Linking numbers, in accordance to (10)**

NAFlex_Circular26_CG, NAFlex_Circular29_1_CG, NAFlex_Circular29_2_CG, NAFlex_Circular30_1_CG, NAFlex_Circular30_2_CG, NAFlex_Circular31_CG, NAFlex_Circular32_CG, NAFlex_Circular33_CG

**Yeast**

- **50µs (frames every 200ps) of the Yeast gene YCL020W**

NAFlex_YCL020W_CG

**Mitochondria**

- **15µs (frames every 1ns) of Human Mitochondrial DNA**

NAFlex_hMDNA_CG

**Implicit Solvent MD**

NAFlex_impSolvMD_CG
