# Supplementary material for: CGeNArate: a sequence-dependent coarse-grained model of DNA for accurate atomistic MD simulations of kb-long duplexes
Source: Nucleic Acids Res. 2024 May 30;52(12):6791–801. doi: 10.1093/nar/gkae444 (PMC11229373; doi:10.1093/nar/gkae444)
Supplement: gkae444_Supplemental_Files [file gkae444_supplemental_files.zip › SI_CGeNArate_30_04_2024.docx]

**Supporting Information for**

**CGeNArate: A Sequence-Dependent Coarse-Grained Model of DNA for Accurate Atomistic MD Simulations of kb-long Duplexes**

David Farré-Gil, Juan Pablo Arcon, Charles A. Laughton and Modesto Orozco*

*Correspondence to M.Orozco: [modesto.orozco@irbbarcelona.org](mailto:modesto.orozco@irbbarcelona.org)

**Supplementary Figures**


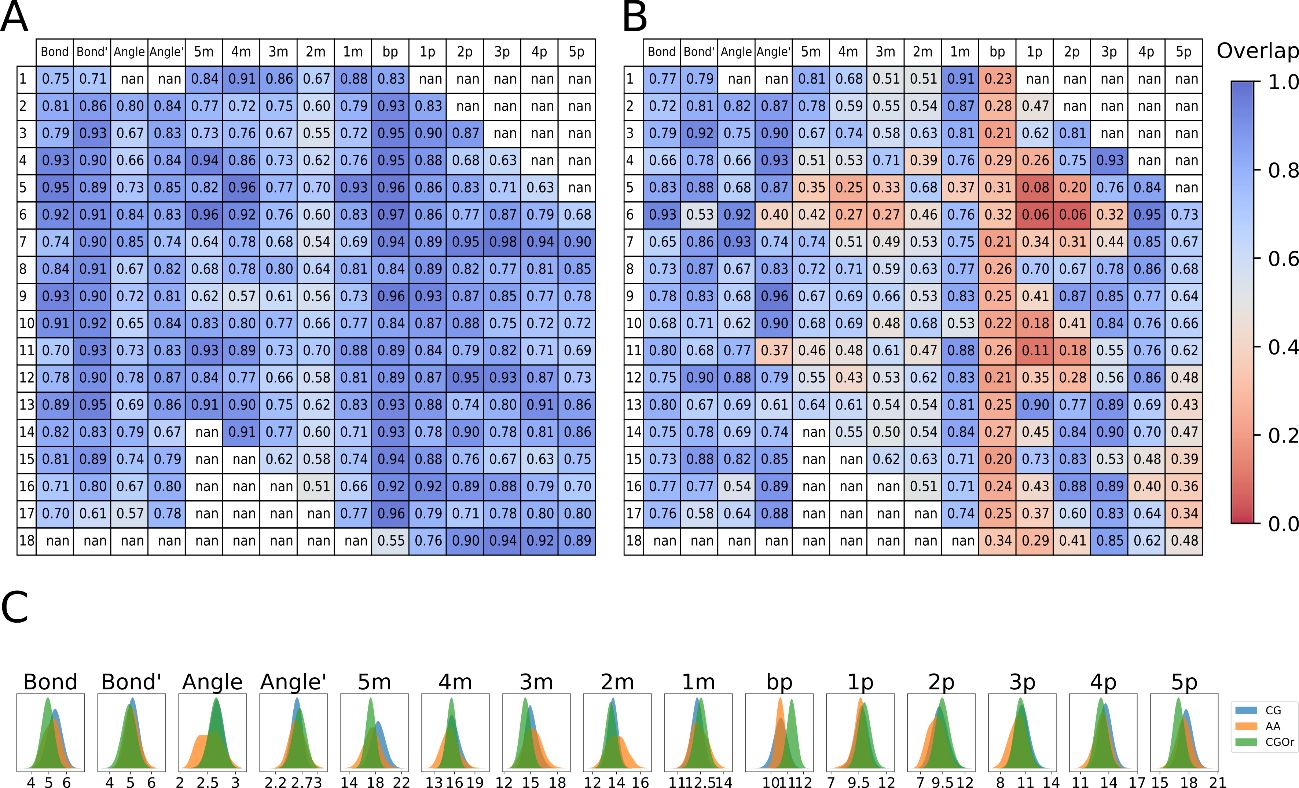


Figure S1. Comparison between distance distributions for the different Hamiltonian terms of the CG Model against AA, before and after optimizing parameters.

A. Distribution overlaps between bonded terms in our refined model vs AA reference.

B. Same as A for pre-refinement model.

C. Detail of Distributions for Bead 8 as an example. (AA = orange, CG before optimization = green, CG after optimization = blue).


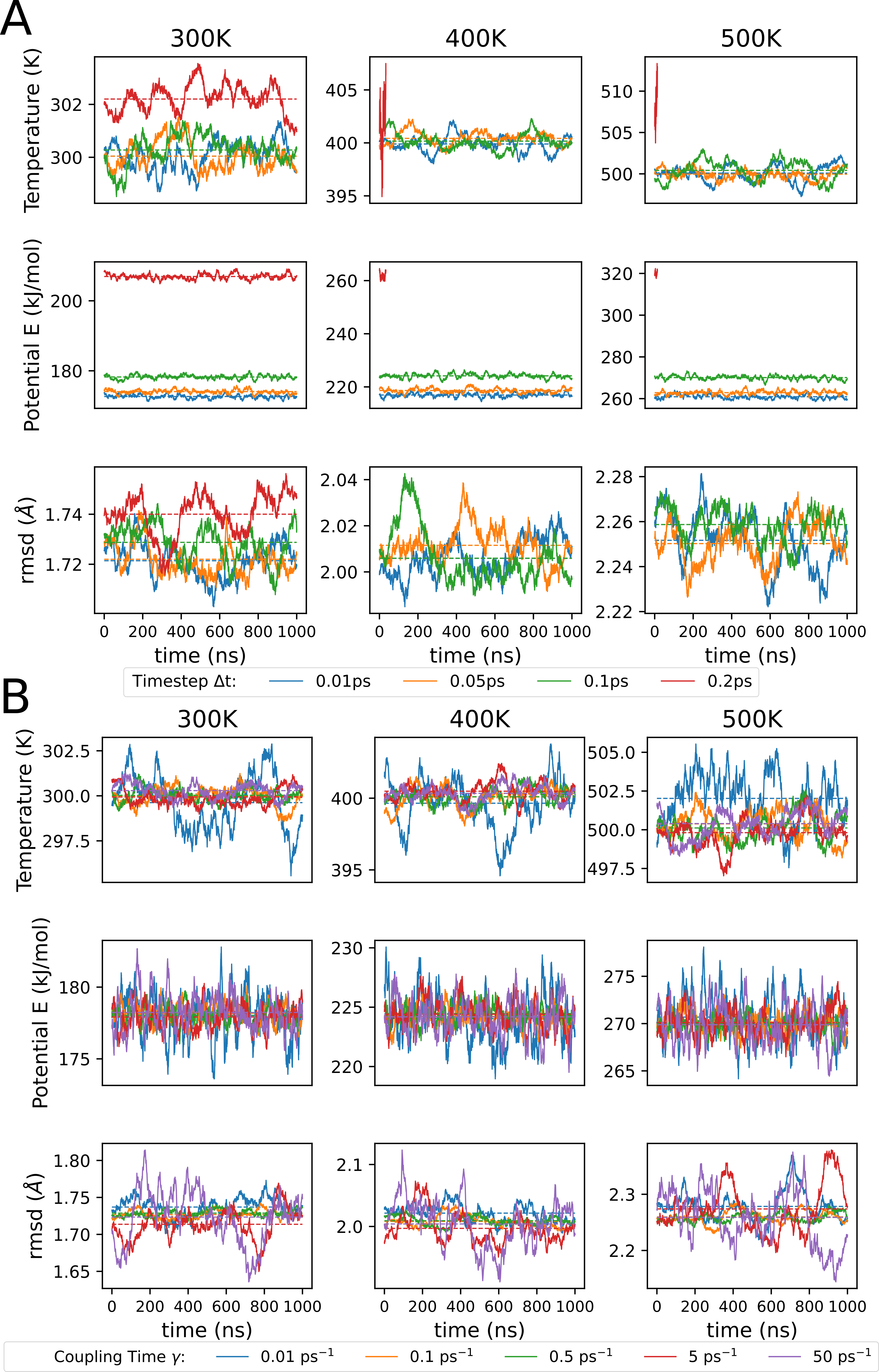


Figure S2. Panel A: Behaviour of the CG model for different integration Timesteps, evaluated for different starting temperatures (300,400,500 K) and recording observed Temperature, Potential Energy and rmsd of C1’ atoms against average CG structure (averages are indicated as a straight line). Panel B: Behaviour of the CG model for different coupling times (the parameter defining the friction coefficient in Langevin equation), evaluated at different temperatures. Note that we use here standard coupling factor 0.5 ps^-1^, but similar results are obtained for other (realistic) coupling times (note the scale of the graphs). Coupling times of 0.01, 0.1, 0.5, 5 and 50 ps^-1^ give friction constants of 0.001, 0.01, 0.05, 0.39 and 0.99, respectively.


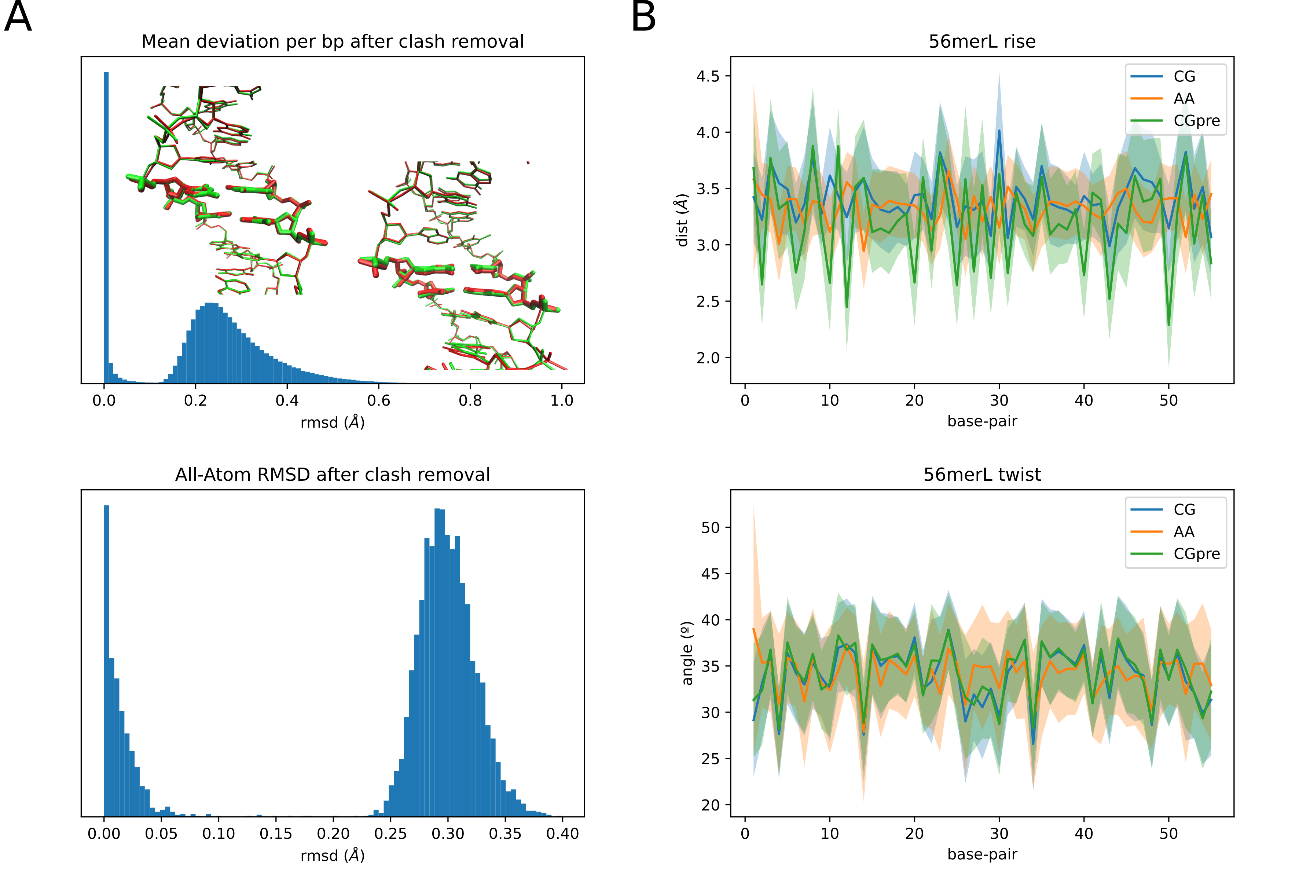


Figure S3. Analysis of the clash removal step on the atomistic reconstruction of the 56-mer simulation.

A. bp-rmsd (up), and whole structure rmsd (down) between pre-minimization and post-minimization structures. Detail of representative structures at average and maximum steric clashes are shown.

B. “rise” (up) and “twist” (down) parameters before (green) and after (blue) minimizing the reconstructed structure. AA (orange) also shown for comparison. Lines represent mean values, and shadows represent one standard deviation.


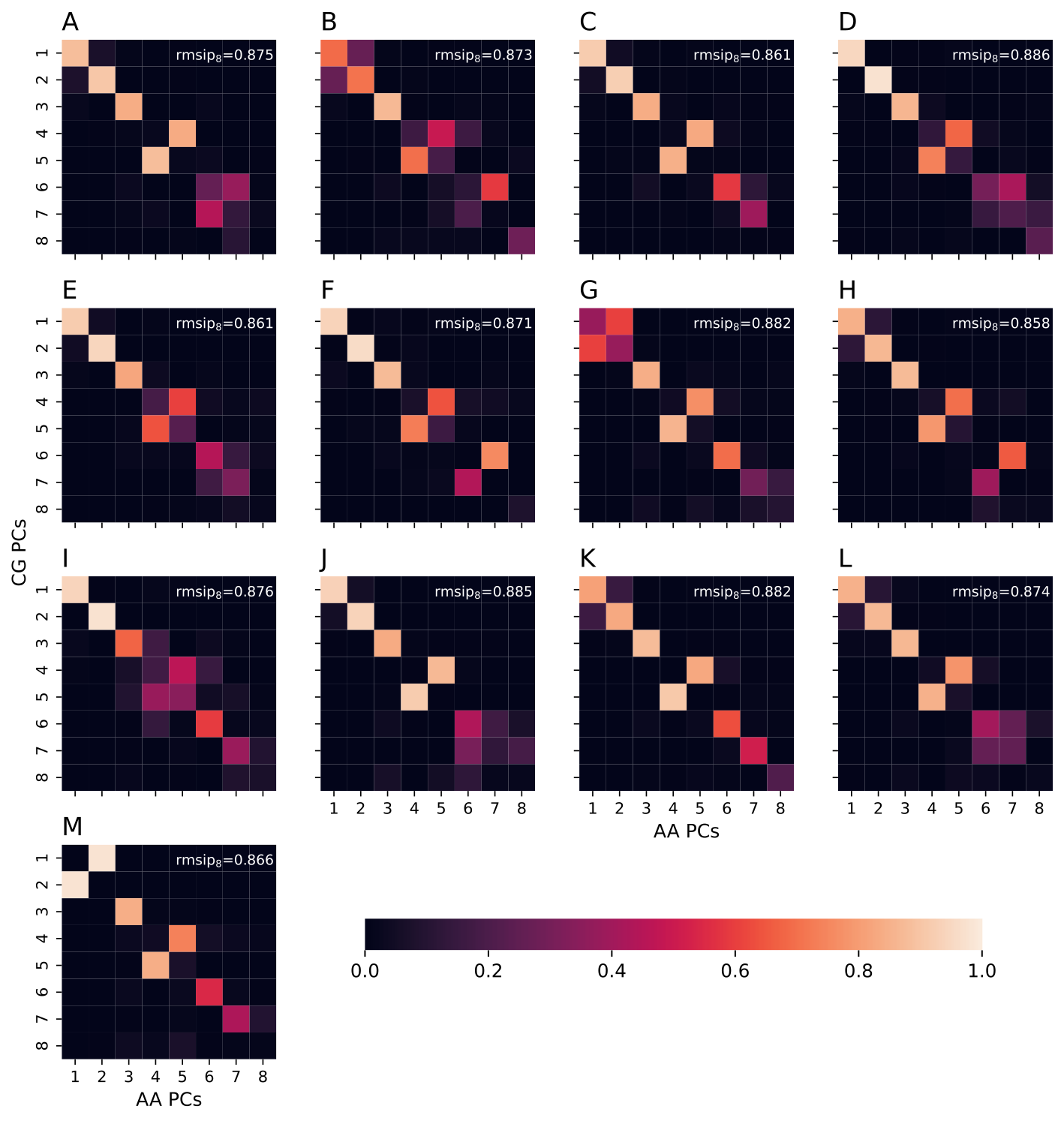


Figure S4. Root mean square inner product matrices between CG and AA Principal Components of trajectories from the training set. Letters A to M correspond to the 13 structures from the miniABC dataset. (See Methods)


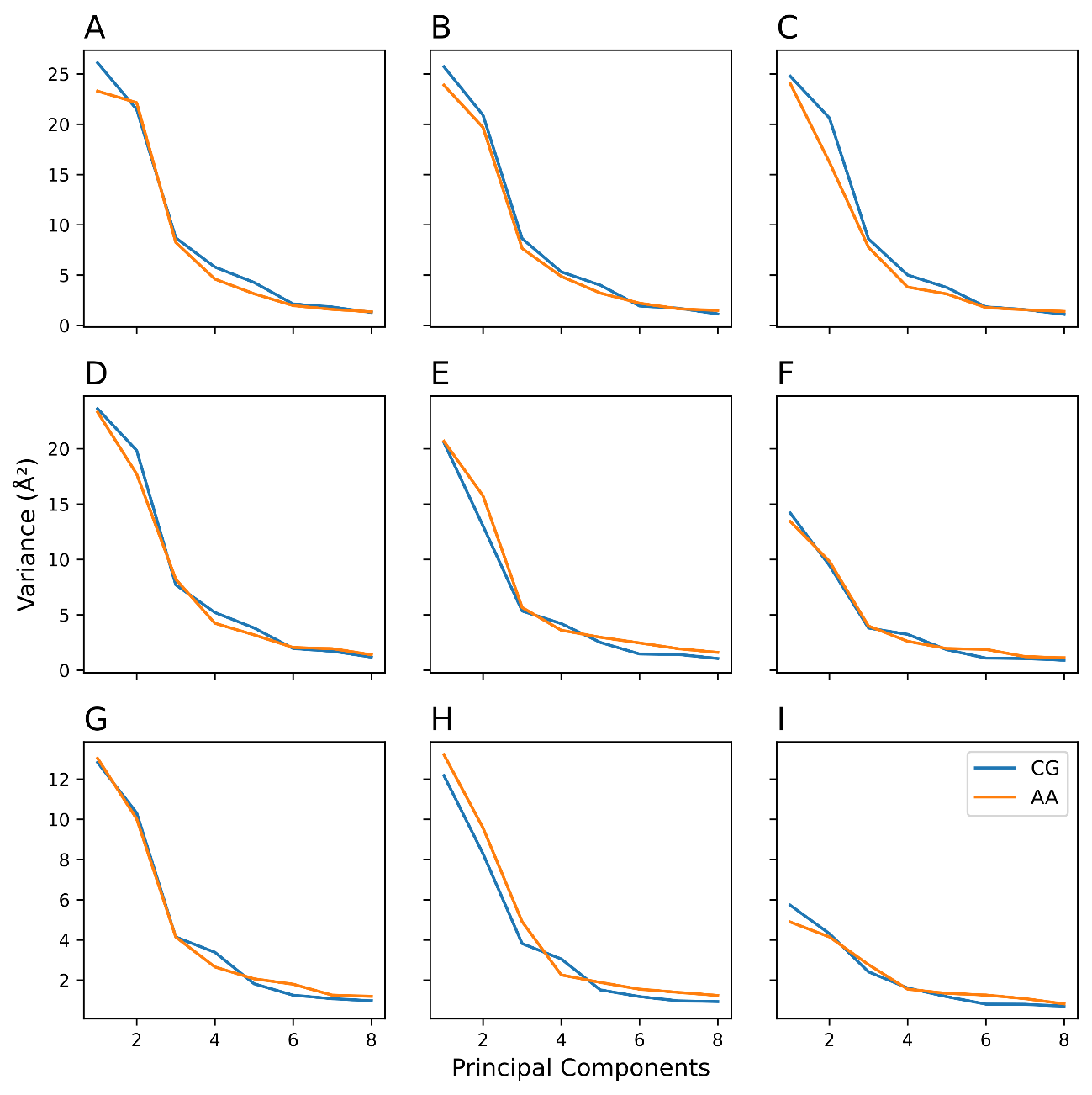


Figure S5. Plot of CG and AA Principal Components of Structures from the testing set, ordered by decreasing variance. Each panel corresponds to simulations of the following structures. A: BigNAsim Code CGTG, B: BigNAsim Code AGCT, C: 1zgw, D: BigNAsim Code AGCG, E: BigNAsim Code CTAG_flex, F: 2lef, G: 1j5n, H: 2m2c, I: 1naj.


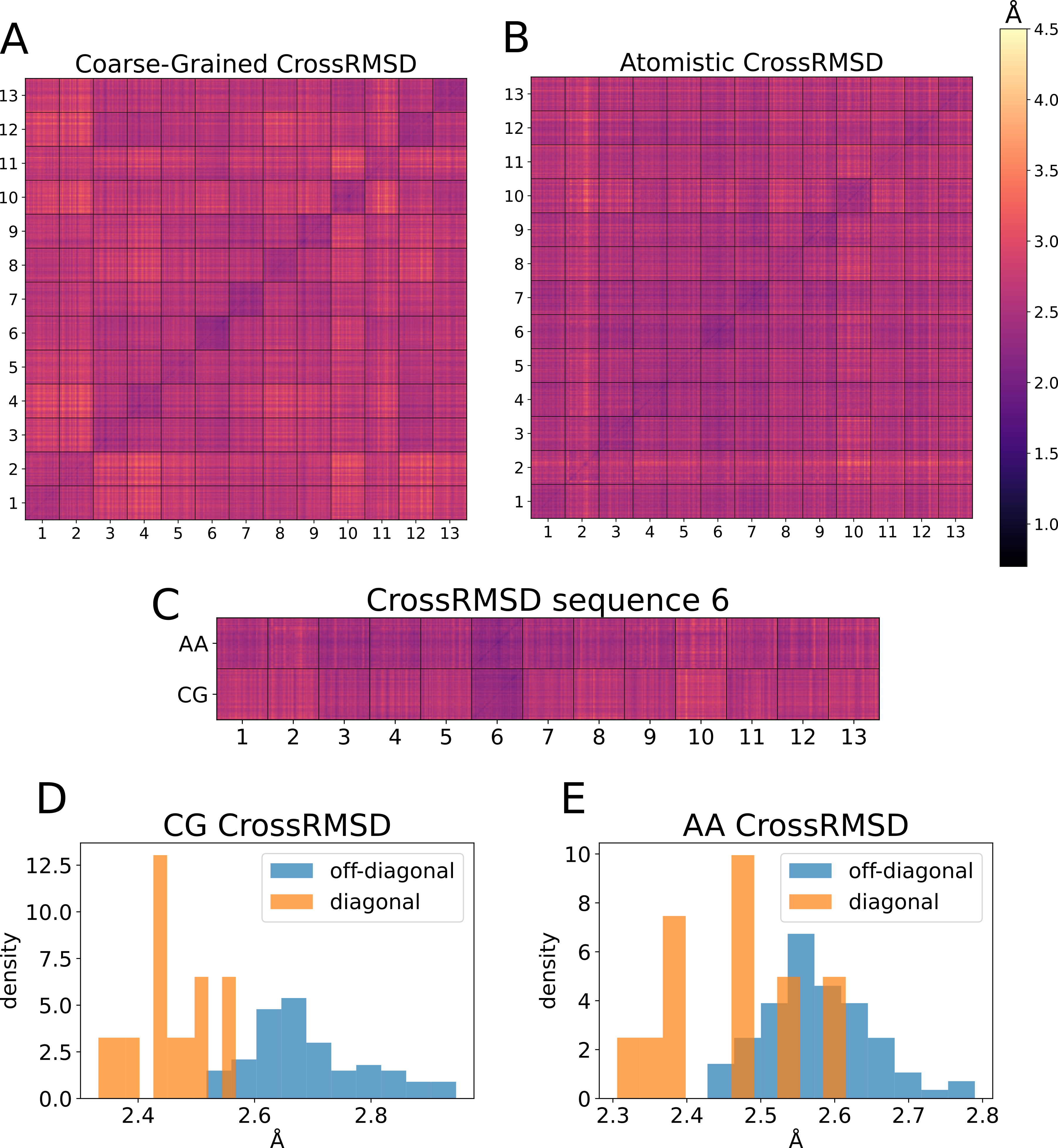


Figure S6. CrossRMSD for CG and AA simulations of the 13 18-mers from the miniABC library

A. CrossRMSD between 500 structures per trajectory of the AA trajectories (All frames against all frames).

B. CrossRMSD between 500 structures per trajectory of the CG trajectories (All frames against all frames).

C. Comparison detail of a row of the matrices from A,B.

D,E. Histogram of average CrossRMSD per sequence pair of the CG (AA) trajectories. Diagonal elements are CrossRMSD between structures of the same sequence, while off-diagonal elements are between different sequences.


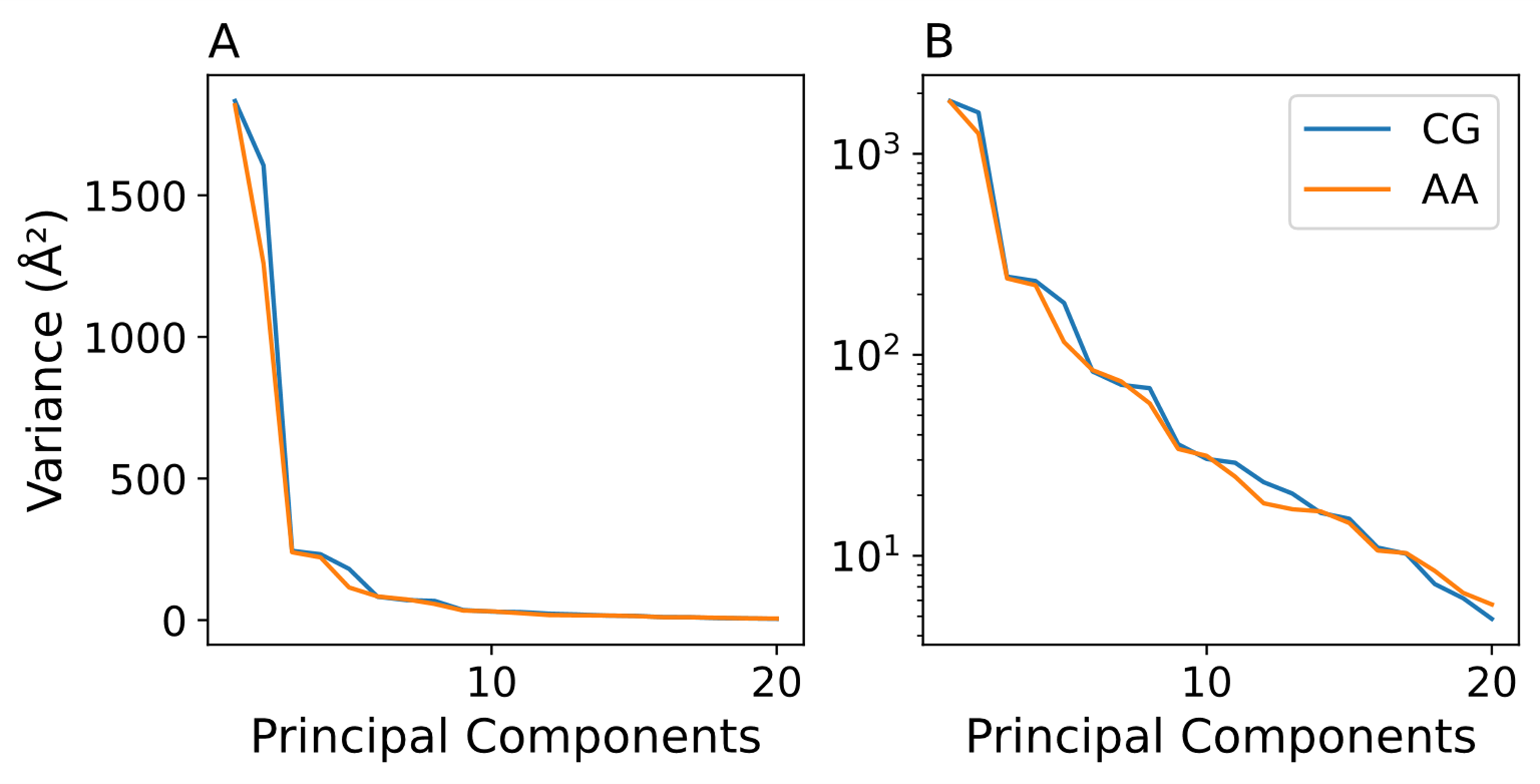
Figure S7. Results from the 56-mer simulations.

A. Variance comparison between CG and AA Principal Components extracted from the respective trajectories, in absolute scale.

B. The same variance comparison, in logarithmic scale, to visualize the correlated variance decay in the 20 PC.


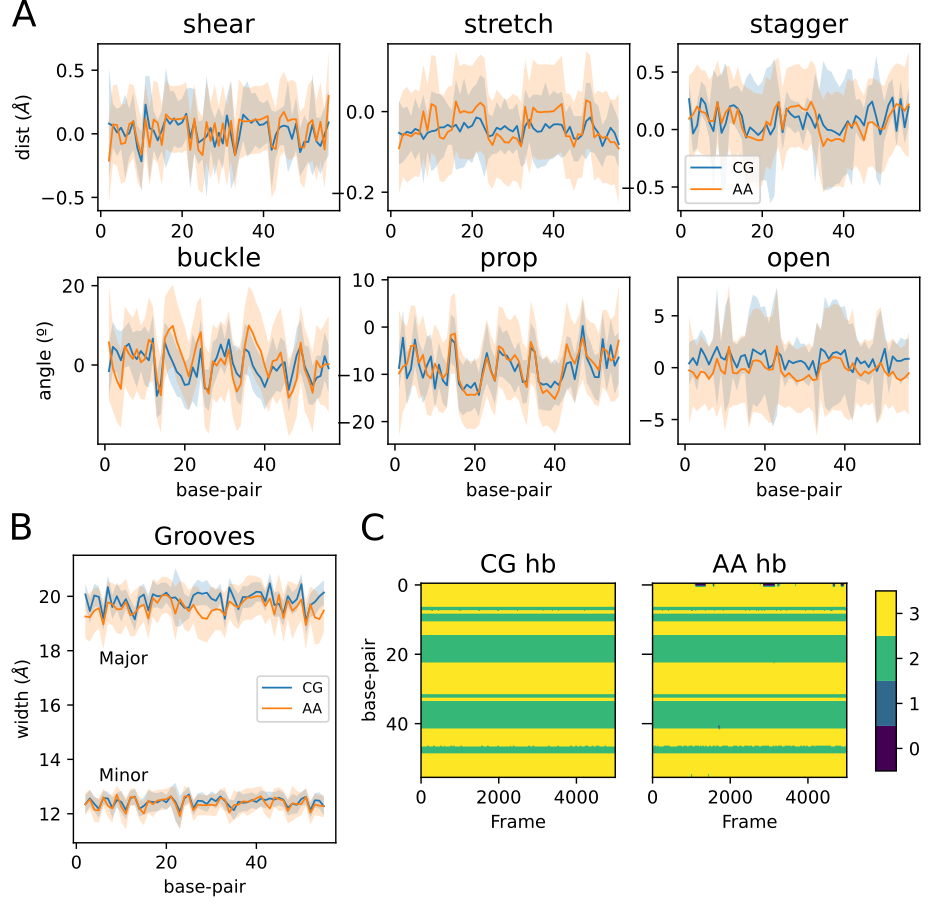


Figure S8. Base pair geometries of the 56-mer simulations

A. Intra base-pair helical parameters for each base-pair step for both CG and AA trajectories. Lines represent mean values, and shadows represent 1 standard deviation.

B. Major and minor groove widths for each base-pair step for both CG and AA trajectories. Lines represent mean values, and shadows represent 1 standard deviation.

C. Presence of hydrogen bonds throughout the simulation for each base-pair. Green bars correspond to AT pairs, while yellow bars correspond to CG pairs.


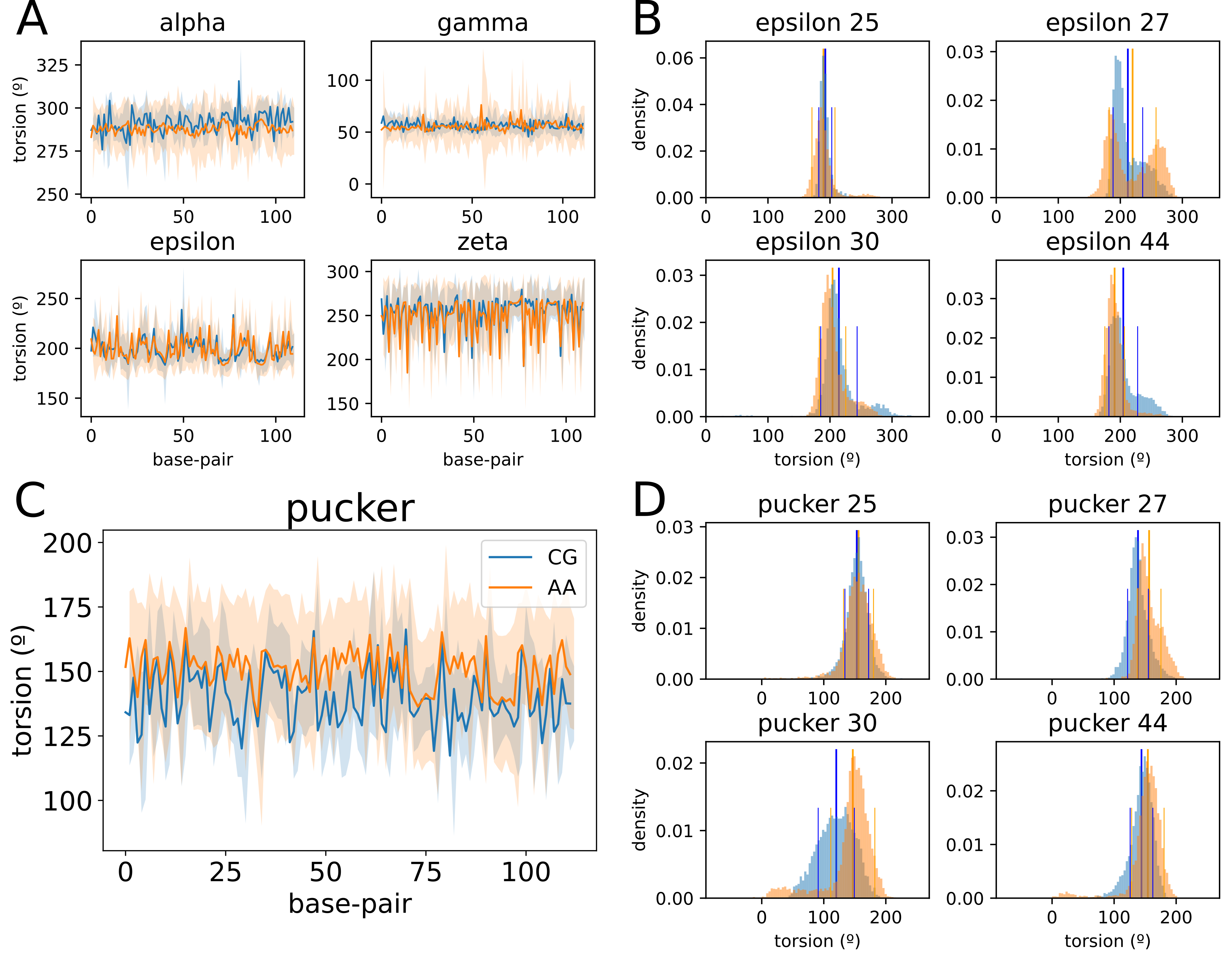


Figure S9. Base pair geometries of the 56-mer simulations

A. Backbone torsion angles for each base-pair step for both CG and AA trajectories. Lines represent mean values, and shadows represent 1 standard deviation.

B. Selection of Epsilon torsion angles showing various conformations across the sequence. Horizontal lines indicate mean values, and 1 standard deviation.

C. Sugar pucker for each base-pair step for both CG and AA trajectories. Lines represent mean values, and shadows represent 1 standard deviation.

D. Selection of Puckering torsion angles showing various conformations across the sequence. Horizontal lines indicate mean values, and 1 standard deviation.

Note the good agreement and the efforts of the back-mapped structures to recover non-harmonic distortions by widening the distributions.


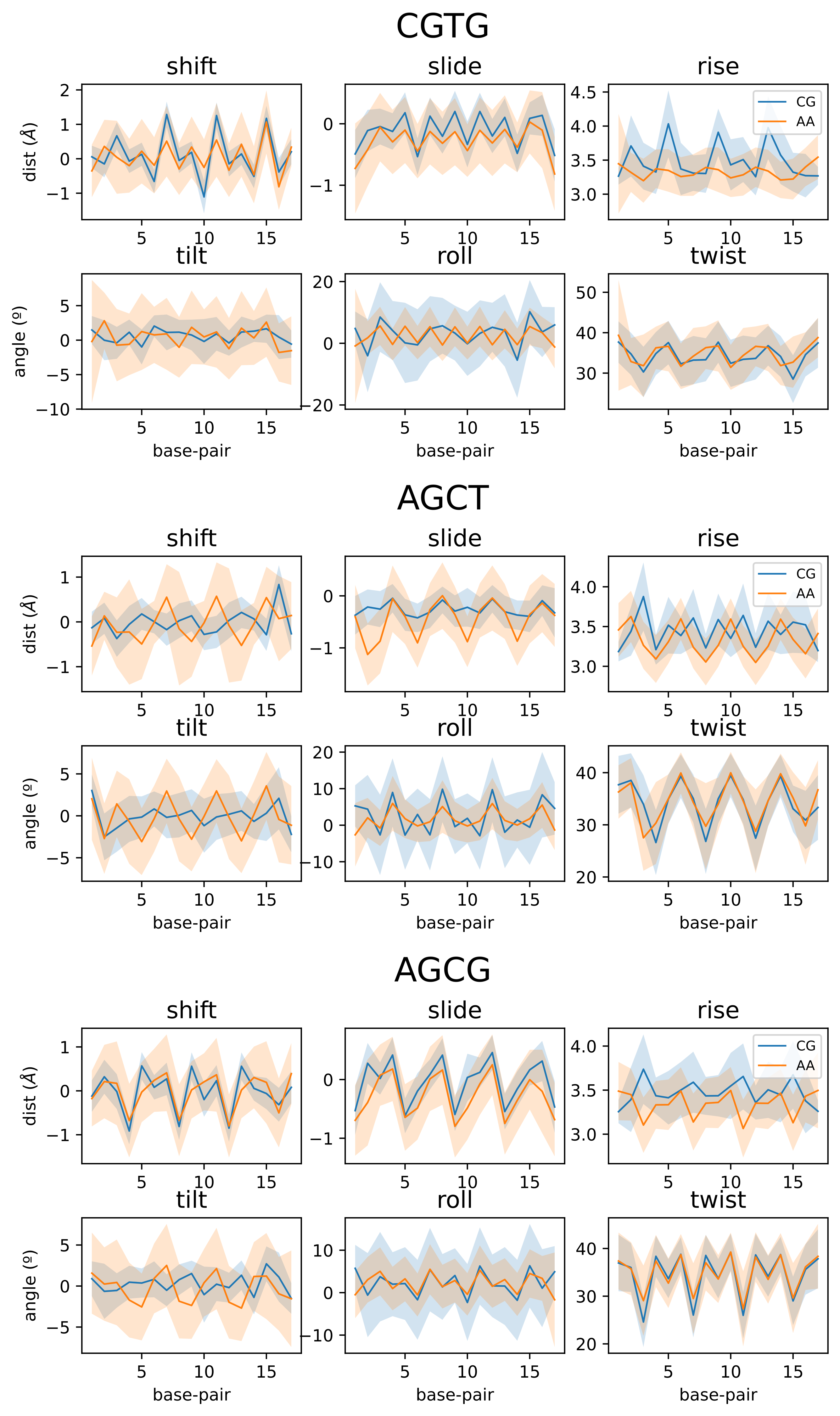


Figure S10. Helical parameters across each base-pair step for CG and AA trajectories of random sequences containing the quoted tetramer. Lines represent mean values, and shadows represent 1 standard deviation.

Each panel corresponds to simulations of the following structures: BigNAsim Code CGTG, BigNAsim Code AGCT, BigNAsim Code AGCG


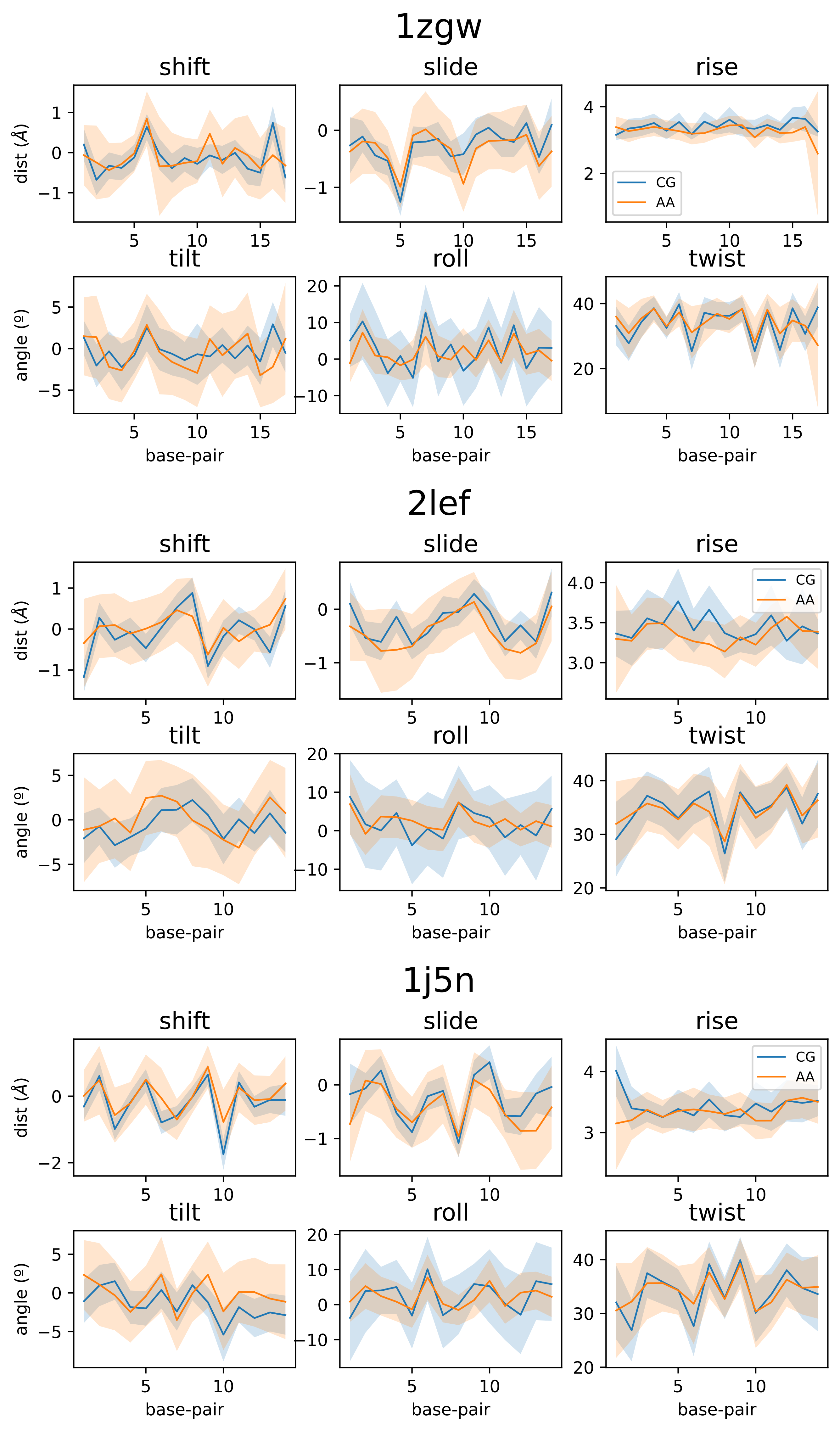


Figure S11. Helical parameter across each base-pair step for CG and AA trajectories obtained for duplexes in PDB for which we know atomistic MD provides good descriptions. Lines represent mean values, and shadows represent 1 standard deviation.

Each panel corresponds to simulations of the following PDB codes: 1zgw, 2lef, 1j5n


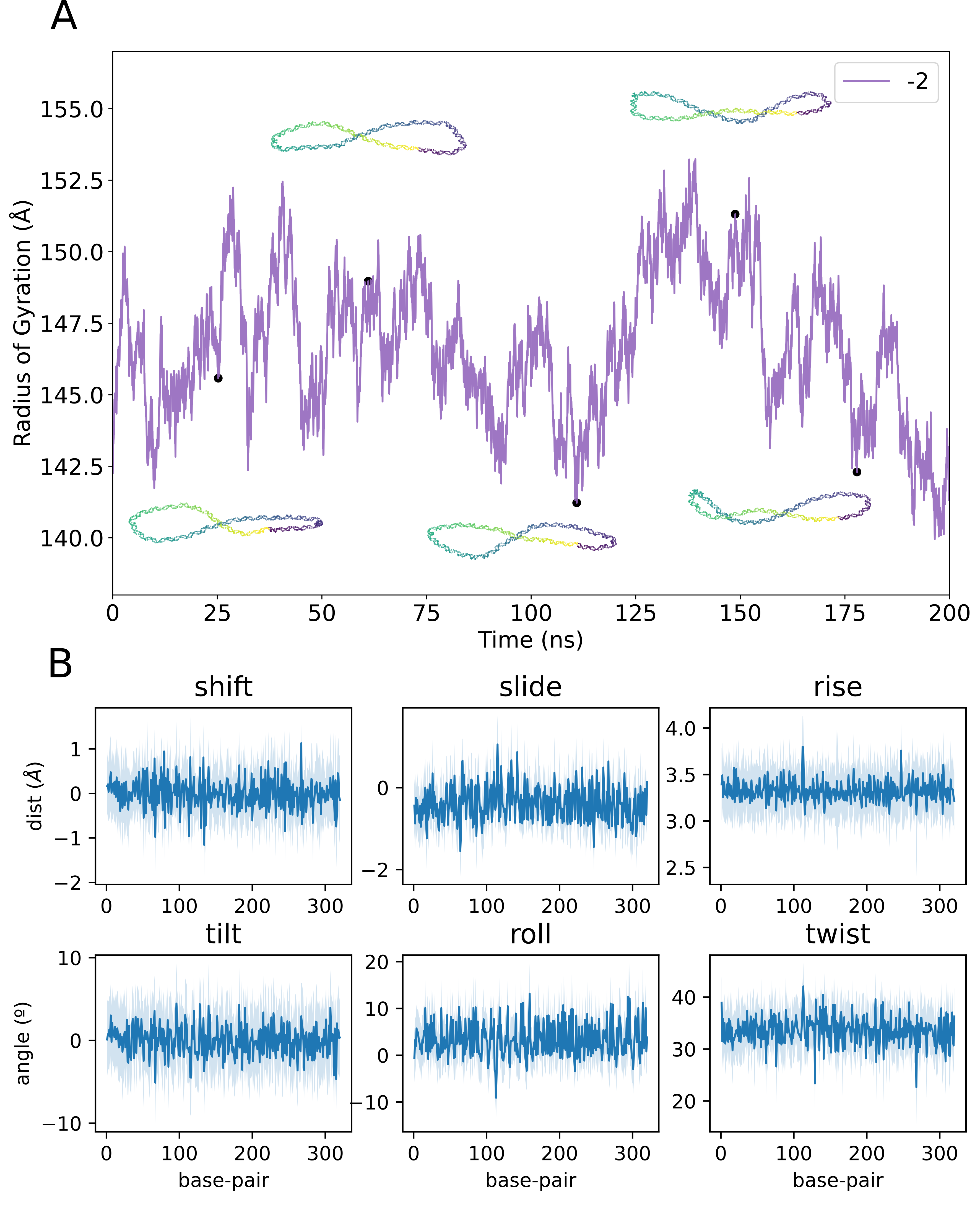


Figure S12. Simulation of the Circle with Linking number 30 (ΔLK=-2)

A. Radius of Gyration across time. Representative structures of the Circle across the simulation are shown. Black points represent the exact time at which the structure was captured. Colouring within the circle accounts for sequence, going from yellow to purple, where it closes back. The structures shown are visualized by projecting the 3D structure to the 2D-plane of best fitting.

B. Helical parameter across each base-pair step for CG trajectory. Lines represent mean values, and shadows represent 1 standard deviation.


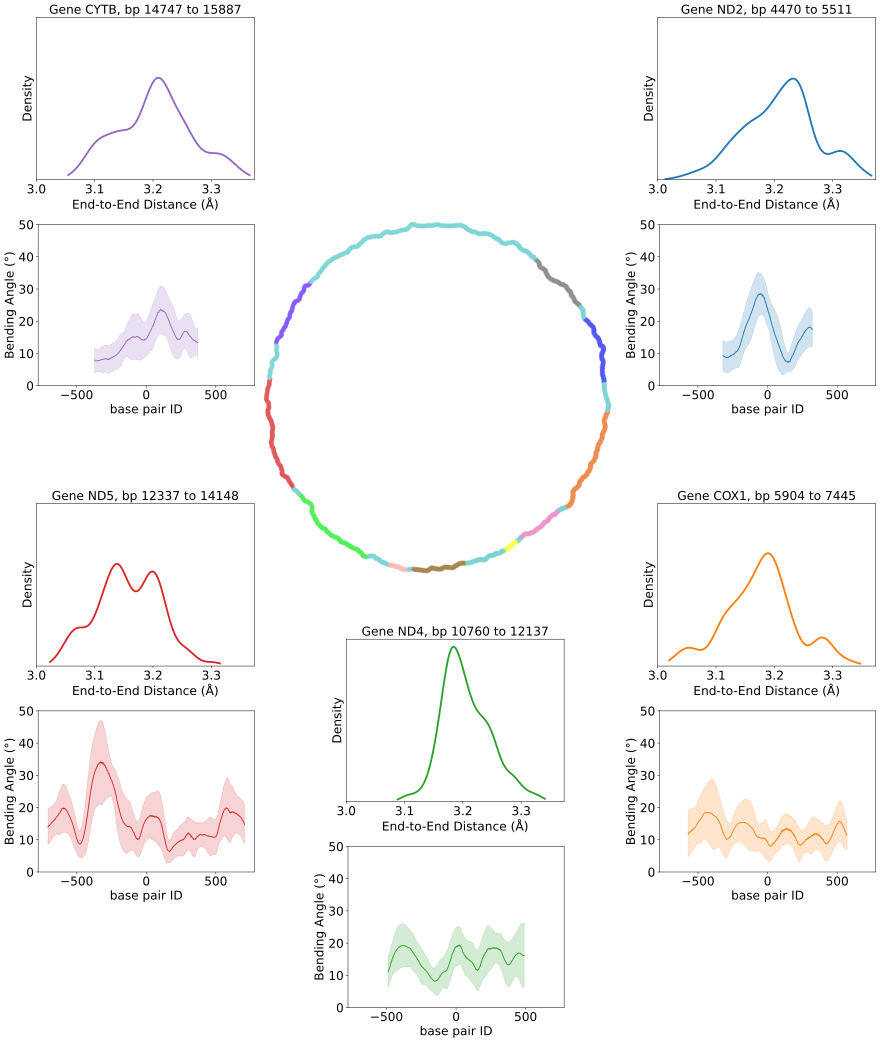


Figure S13. Human mitochondrial DNA simulation. Plots are shown for the five longest coding genes of the mitochondrial DNA, color-coded in both the graph and structure. The top plot displays the distribution of the (normalized) 3D distance between the two ends of each gene, while the bottom plot shows the bending angle of the gene along the sequence, centred at the midpoint of the gene. Lines represent mean values, and shadows represent one standard deviation. Simulations were performed for 15 microseconds.


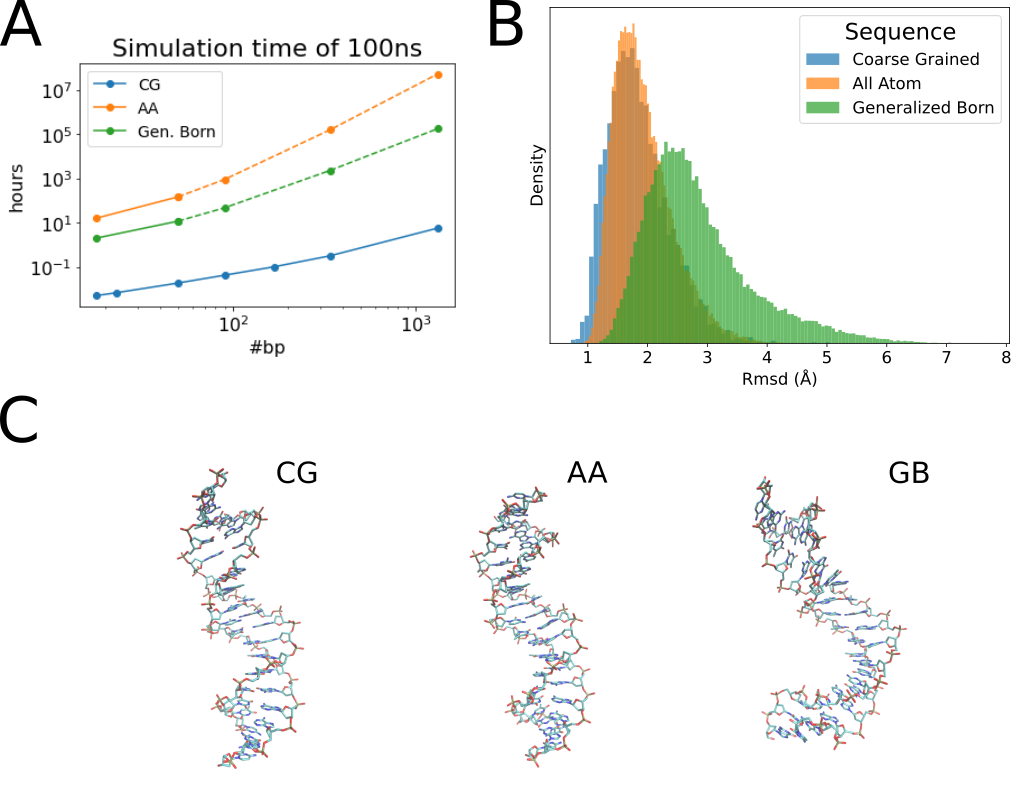


Figure S14. Performance of our CG model (blue) compared with atomistic simulations with explicit (orange) and implicit GB/SA solvent (green).

A. CPU time for duplexes of different length (dashed lines indicate predicted time of a comparable AA simulation, obtained as a linear extrapolation of the available datapoints and CG time)*.* Computations are performed on the same nodes, with the following specs: Intel(R) Xeon(R) CPU E5-2670 0 @ 2.60GHz, 32GB RAM. AA data for very long systems are approximated based on publicly available benchmarks based on the size of the predicted simulation box.

B. Histogram of all (heavy) atoms RMSd (in Å) with respect to the canonical B-form (excluding terminal bases).

C. Structures obtained at the end of a 500 ns simulation. All structures are aligned to the same reference. The provided CG structure is back-mapped from the C1' model

**Supporting Information Text**

**Extended Methods**

**Remote term specifications**

The remote term is divided as follows:

$$E_{remote}=E_{LJ}+E_{ele} (7)$$

The “remote steric” term treated by a Lennard-Jones potential avoids steric classes between remote segments of DNA (Eq. 8), or in future implementations with interacting particles.

$$E_{LJ}=4\epsilon_{LJ}\left( \left( \frac{\sigma}{r} \right)^{12}-\left( \frac{\sigma}{r} \right)^{6} \right) (8)$$

where $\epsilon_{LJ}$ and $\sigma$ accounts for the hardness of the interaction and the size of the beads, respectively.

The “remote electrostatic contribution” is computed using Debye-Hückel potential as described by Beard and Schlick(1) (Eq.9)

$$E_{ele}=\frac{{q_{1}q}_{2}}{\varepsilon\varepsilon_{0}r}e^{-\kappa r} (9)$$

where q_i_ are set to the charge of one electron, r is the distance $\varepsilon_{0}$ is vacuum permittivity, $\varepsilon$ is the dielectric constant relative to vacuum and κ is the inverse Debye length, which for an electrolyte takes the form shown in Eq. 10:

$$\kappa=\sqrt{\left( \frac{\varepsilon\varepsilon_{0}k_{B}T}{2{q_{e}}^{2}I} \right)} (10)$$

where $k_{B}$ is Boltzmann constant, T is the temperature, q_e_ is the charge of one electron, and I stands for the ionic strength of the electrolyte.

**Parameter fitting at tetranucleotide level**

Tetramer-level fitting was performed from microsecond-long trajectories (5x10^4^ frames each) of the thirteen 18-mer duplexes from the miniABC dataset (2)(2)(2), which contain information on the unique 136 tetramers, which were explored for 1 microsecond unrestricted MD simulation in explicit water using 2 fs integration step, periodic boundary conditions, PARMBSC1 and state of the art protocols (2). For those tetramers appearing more than once in the miniABC database, the different ensembles were aggregated. Atomistic trajectories were then projected to the reduced C1’ space (i.e., 8 beads for tetramer) defining the 16 observables to fit (4 bond distances, 8 fan distances, and 4 angles) and collecting distributions from them. Refinement of the 16x3 parameters was done for each tetramer independently. We then use the COBYLA algorithm using as seeding parameters a series of equilibrium force constant {d_0_, K_o_} obtained by linear polynomial fitting of each (1D) distribution following the form of eq. (3). A CG simulation was then launched with these guess parameters and the associated distributions were compared to the atomistic ones. The deviation leads the COBYLA algorithm (see Methods) to suggest a new set of parameters {d_1_, K_1_}, repeating the process until a final set of parameters {d_n_, K_n_} were obtained. Restrictions were introduced to avoid unphysical fittings. For example, the 2^nd^ and 4^th^ order terms were forced to be positive and global function should have only one minimum. As shown in Suppl. Figure S1 the process is fast, efficient, less prone to convergence problems than previously suggested methods.

As described in Methods the long-range interactions were fitted after the short-range 4mer terms were refined assuming no sequence-dependence beyond the tetramer and using a long 40-mer duplex simulation as reference. The refinement procedure was identical to that described above for the short-range terms.

**Evaluation of Back-mapping method**

The deluge of structural parameters recovered from the back-mapped trajectories show that these are largely comparable to trajectories obtained from AA MD.

One of the key issues that can be observed when looking at these plots, is the fact that mean values are recovered within small error ranges in almost all cases, whereas the variability of the parameters is not equally recovered in all cases. This is mostly explained by two factors.

The nature of the CG simulations and the back-mapping process, take into account certain assumptions and sometimes focus on relatively static data. The CG simulations are very simplified at the base level, and therefore don’t characterize all possible configurations for all possible parameters. For instance, Tilt is largely independent of the relative positions of C1’ atoms, as the backbone is largely unaffected then this parameter changes. This is not the case, for instance, for Twist, which is majorly dependent on the backbone C1’ positions. Then, these degrees of freedom are not all equally dealt with in the back-mapping. Because the reconstruction works by similarity to atomistic structures, it will recover structures largely around the mean parameter. The parameters that are more precisely recovered from the C1’ representation will have a more accurate distribution, that comes form the intrinsic variability in the simulations, whereas those parameters that are not as characterized will have less accurate distributions. The back-mapping is incorporating the variability in different stages and leads to some very rigid assumptions, that require later overrelaxation to account for the total variance.

A second effect that can clearly be observed in the Density plots provided for epsilon and pucker, is that not all distributions are comparable in global shape. Mostly this can be cause by bimodality, or high skewness of the distributions. When simulating and recovering the trajectories there are simplifications intrinsic to CG models that remove some of the ‘bumpyness’ of real Atomistic potentials and leads to somewhat over-smoothed potentials. While this is largely accounted for in the sequence-dependent tetranucleotide modelling, the back-mapping has a larger tendency to focus on averages and can lead to inexact estimations. This is somewhat unavoidable, and reinforces the need for an energy-based minimization to differentiate into the modes of more complex distributions.

**Sequences used for validation**

For validating the model, we used several sets of atomistic simulations not considered during the training. All of them are available in the BigNAsim database (see main text). They include the following systems :

- 56mer duplex (ID: NAFlex_56merL):

d(CGCCGGCAGTAGCCGAAAAAATAGGCGCGCGCTCAAAAAAATGCCCCATGCCGCGC)

- a variety of 16-mers duplexes with repetitive sequences:

ID NAFlex_CGTG, sequence: d(CGTGCGTGCGTGCGTG)

ID NAFlex_AGCT, sequence: d(GCCTAGCTAGCTAGCTGC)

ID NAFlex_AGCG, sequence: d(GCCGAGCGAGCGAGCGGC)

ID NAFlex_CTAG_flex, sequence: d(CGCTCTCTAGAGAGGC)

- different simulations launched from experimental structures with the following PDB codes:

2lef (ID: NAFlex_2lef)

1j5n (ID: NAFlex_1j5n)

1zgw (ID: NAFlex_1zgw)

1bna (ID: NAFlex_DDD_II)

1naj (ID: NAFlex_DDD_II_1)

2hkb (ID: NAFlex_2hkb)

2k0v (ID: NAFlex_2k0v)

2l8q (ID: NAFlex_2l8q)

2m2c (ID: NAFlex_2m2c)

These duplexes were simulated for simulation periods in the range 200 ns- 1 microsecond in explicit solvent (TIP3P or SPC/E water models, Dang ion parameters) using PARMBSC1 force field and simulation conditions as in ref (2).

**Implicit Solvent MD**

MD in implicit solvent was performed in AMBER using the Hawkins, Cramer, Truhlar(3, 4) pairwise generalized Born model, with parameters described by Tsui and Case(5). The exterior dielectric constant was set to 78.5 (relative to vacuum), while the interior dielectric constant of the molecule of interest was set to 1. The salt concentration was adjusted to 0.15M, based on the Debye-Hückel limiting law for ion screening of interactions(6). Bonds involving hydrogen atoms were constrained with the SHAKE algorithm, and all non-bonded interactions were calculated (10,000 A cutoff, effectively infinite). The temperature was kept around 298 K using the Langevin thermostat with a collision frequency of 1 ps-1. No periodic boundaries were set.

**MD Simulation Time Estimates**

Simulation times for MD simulations have a well-studied correlation with the number of (heavy) atoms. Therefore, for estimating simulation times of large systems, our main concern is determining the number of atoms of such systems. The atoms in the canonical DNA duplex are 41 per base-pair. For a linear DNA, the box size should be the size of the duplex, i.e. 3.3Å x bp in rise, and 24Å width, plus at least 15Å distance from the duplex to the box. This conservative estimate gives a box of 54x54x(40+3.3*bp) Å^3^, with 0.03345 water molecules(1 heavy atom) per Å^3^. For physiological conditions (0.15M) and neutralizing the DNA, there will also be (2*bp+2*0.002711*waters) ions.

**Extended description of metrics**

**Metrics for evaluation of the quality of CG ensembles**

We use standard Cartesian descriptors of divergence between a trajectory and a reference structure (the global RMSd and the residue fluctuation RMSf). Helical parameters were determined as described in Curves+ and using the associated programs (7).

The similarity between distances/angles in atomistic and CG simulations was determined by the distribution overlap defined as:

$$Overlap\left( f,g \right)=\int min\left( f\left( x \right),g\left( x \right) \right)dx$$

where f(x), g(x) are the two distributions. Note that as:

$$\int f\left( x \right)dx=\int g\left( x \right)dx=1$$

the overlap goes from 0 (when the distributions share no values) to 1 (when the distributions are exactly the same)

The bending angle at a given position of the polymer was computed as that defined by the vectors originating in that point and ending in two points separated by window distance “s”. This calculation can be performed for all points in a polymer, leading to a bending angle profile that represents regions of higher flexibility within the polymer. Note that the bending angle of base-pair i is computed as the angle between vectors (bp_i_-bp_i-s_) and (bp_i+s_-bp_i_), with base pair being defined as the middle point between the paired beads. Finally, the radius of gyration was computed as the root mean square distance of the beads to the centre of mass of the DNA duplex.

The aspect ratio is defined as the proportion between the width and the height of a rectangular prism enclosing the DNA duplex, smaller the value more elongated is the duplex.

**Global flexibility descriptors**

End-to-end: The end-to-end vector was determined taking as reference the midpoint of the two terminal bp as the end of the polymer:

$$EndtoEnd=dist\left( \frac{p_{1}+p_{2n}}{2},\frac{p_{n}+p_{n+1}}{2} \right)=\left\| \frac{p_{1}+p_{2n}}{2}-\frac{p_{n}+p_{n+1}}{2} \right\|$$

The persistence length (PL) is defined as the length over which correlations in the direction of the tangent are lost as defined by the SerraNA program (Velasco-Berrelleza et al., 2020).

Principal Component Analysis (PCA) was used to define the essential deformation space of a given duplex DNA. Note that comparison of the associated eigenvectors/eigenvalues of two simulations (in our case CG and atomistic) provides direct information on how similar the essential deformation modes captured by the two methods are. To compare the nature of the deformations we look at the root mean square inner product matrix (9) defined as:

$$\sqrt{\frac{1}{n}\sum_{i=1}^{n} \left( \sum_{j=1}^{n} \left( PC1_{i}\cdot PC2_{j} \right)^{2} \right)}$$

where the sum extends for a given set of n essential movements (eigenvectors, PC) of simulation i or j. In our case, we considered a fixed n=8 for most of our simulations, which amounts to 80-90% of the total variance. For the 56mer we consider n=20, accounting for around 95% of the total variance.

We also take advantage of the intermediate square inner product matrix

$${M_{ij}=\left( PC1_{i}\cdot PC2_{j} \right)}^{2}$$

which allows us to visualize similarities across the eigenvectors. The closer the matrix resembles a diagonal, the better the similitude between the trajectories.

**Supplementary Legends**

**Movie S1 yeast8microsec.mp4**

Movie showing the movement and bending of gene YCL020W of *Saccharomyces cerevisiae*, from a straight structure to a relaxed conformation. Movie shows the first 8µs of Simulation.

**Movie S2 mitochondria.mp4**

Movie showing the movement of the Human Mitochondrial DNA at different scales, including the reconstructed atomistic trajectory, giving an overview of the multiscale nature of the system. Movie shows 15 μs of simulation.

**Dataset S1**

Parameter set for the bonded interactions of the Hamiltonian.

Files MyD.txt, MyDLong.txt contain Equilibrium distances for *4mer* and *distant* interactions, respectively.

Files MyK.txt, MyKLong.txt contain Force constants for *4mer* and *distant* interactions, respectively.

These files can be found in the following directory in the submission gitlab <https://mmb.irbbarcelona.org/gitlab/dfarre/cgenarate-materials/-/tree/main/CGModelCode/input>

**Dataset S2**

Pickled GLIMPS models for all-atom rebuilding of Coarse-Grained DNA.

Files transformer_backbone.pickle, transformerA.pickle, transformerC.pickle are the GLIMPS models which rebuild the backbone, AT basepairs and CG basepairs, respectively.

These files can be found in the following directory in the submission gitlab

<https://mmb.irbbarcelona.org/gitlab/dfarre/cgenarate-materials/-/tree/main/GLIMPS>

**Dataset S3**

Simulations obtained with CGeNArate of the following sequences:

- 1µs (frames every 20ps) for each of the 13 miniABC sequences (2)
- 1µs (frames every 20ps) for each of the 14 sequences used for validation (as described in Supplementary methods)
- 1µs (frames every 20ps) for Circular DNA with different Linking numbers, in accordance to (10)
- 50µs (frames every 200ps) of the Yeast gene YCL020W
- 15µs (frames every 1ns) of Human Mitochondrial DNA

These files are to be published in a public MD repository (such as BigNAsim). Due to the large volume of data, it is not feasible to make available all simulated trajectories at submission time in a private manner. Nonetheless, reduced trajectories can be made available on request.

**Software S1**

The programs provided are the following:

To simulate linear DNA from a starting pdb structure, *CGeNArate.exe*, with settings file *simulationssettings.txt*, and run by executing *run.sh*, which reads the relevant name files from an appropriate *namesfile****.dat.*

To simulate circular DNA from a starting pdb structure, *CGeNArateCircular.exe*, with settings file *simulationssettingsCircular.txt*, and run by executing *Circularrun.sh*, which reads the relevant name files from an appropriate *namesfile****.dat.*

To rebuild a simulated Coarse-Grained DNA, *Rebuild_nmer.py*. The input and output files have to be edited in the first few lines of the python script.

To generate the starting structures: *fdhelix.c*, containing a simplified version of the original code by David A. Case., and *MitochondriafromLine.py*, a script to convert linear DNA into circular DNA with a specified supercoiling.

All these files can be found in the submission gitlab

<https://mmb.irbbarcelona.org/gitlab/dfarre/cgenarate-materials>

**Supplementary References**

1. Beard,D.A. and Schlick,T. (2000) Modeling Salt-Mediated Electrostatics of Macromolecules: The Discrete Surface Charge Optimization Algorithm and Its Application to the Nucleosome.

2. Dans,P.D., Balaceanu,A., Pasi,M., Patelli,A.S., Petkevičiūtė,D., Walther,J., Hospital,A., Bayarri,G., Lavery,R., Maddocks,J.H., *et al.* (2019) The static and dynamic structural heterogeneities of B-DNA: extending Calladine–Dickerson rules. *Nucleic Acids Res*, **47**, 11090–11102.

3. Hawkins,G.D., Cramer,C.J. and Truhlar,D.G. (1996) Parametrized Models of Aqueous Free Energies of Solvation Based on Pairwise Descreening of Solute Atomic Charges from a Dielectric Medium. *J Phys Chem*, **100**, 19824–19839.

4. Hawkins,G.D., Cramer,C.J. and Truhlar,D.G. (1995) Pairwise solute descreening of solute charges from a dielectric medium. *Chem Phys Lett*, **246**, 122–129.

5. Tsui,V. and Case,D.A. (2000) Theory and applications of the generalized born solvation model in macromolecular simulations. *Biopolymers*, **56**, 275–291.

6. Srinivasan,J., Trevathan,M.W., Beroza,P. and Case,D.A. (1999) Application of a pairwise generalized Born model to proteins and nucleic acids: inclusion of salt effects. *Theoretical Chemistry Accounts: Theory, Computation, and Modeling (Theoretica Chimica Acta)*, **101**, 426–434.

7. Lavery,R., Moakher,M., Maddocks,J.H., Petkeviciute,D. and Zakrzewska,K. (2009) Conformational analysis of nucleic acids revisited: Curves+. *Nucleic Acids Res*, **37**, 5917–5929.

8. Velasco-Berrelleza,V., Burman,M., Shepherd,J.W., Leake,M.C., Golestanian,R. and Noy,A. (2020) SerraNA: a program to determine nucleic acids elasticity from simulation data. *Physical Chemistry Chemical Physics*, **22**, 19254–19266.

9. Pérez,A., Blas,J.R., Rueda,M., López-Bes,J.M., De La Cruz,X. and Orozco,M. (2005) Exploring the essential dynamics of B-DNA. *J Chem Theory Comput*, **1**, 790–800.

10. Pyne,A.L.B., Noy,A., Main,K.H.S., Velasco-Berrelleza,V., Piperakis,M.M., Mitchenall,L.A., Cugliandolo,F.M., Beton,J.G., Stevenson,C.E.M., Hoogenboom,B.W., *et al.* (2021) Base-pair resolution analysis of the effect of supercoiling on DNA flexibility and major groove recognition by triplex-forming oligonucleotides. *Nat Commun*, **12**.
